# Supplementary material for: Nano- to macro-scale control of 3D printed materials via polymerization induced microphase separation
Source: Nat Commun. 2022 Jun 22;13:3577. doi: 10.1038/s41467-022-31095-9 (PMC9217958; doi:10.1038/s41467-022-31095-9)
Supplement: Supplementary file 1 — Supplementary Information [file 41467_2022_31095_MOESM1_ESM.pdf]

Supplementary Information

Nano- to macro-scale control of 3D printed materials via polymerization induced microphase separation

Valentin A. Bobrin[1], Yin Yao[2], Xiaobing Shi[1], Yuan Xiu[1], Jin Zhang[3*], Nathaniel Corrigan[1, 4*], and Cyrille Boyer[1, 4*]

[1]Cluster for Advanced Macromolecular Design, School of Chemical Engineering, University of New South Wales, Sydney, NSW 2052, Australia

[2]Electron Microscope Unit, Mark Wainwright Analytical Centre, University of New South Wales, Sydney, NSW 2052, Australia

[3]School of Mechanical and Manufacturing Engineering, University of New South Wales, Sydney, NSW 2052, Australia

[4]Australian Centre for Nanomedicine, School of Chemical Engineering, University of New South Wales, Sydney, NSW 2052, Australia

*Email: jin.zhang6@unsw.edu.au, n.corrigan@unsw.edu.au, cboyer@unsw.edu.au

Materials and Supplementary Methods

Materials

Unless otherwise stated, all chemicals were used as received. The solvents were of either HPLC or AR grade; these included acetonitrile (RCI Labscan Limited, RCI Premium) and *N*,*N*-dimethylacetamide (DMAc, RCI Labscan Limited, HPLC). Aluminium oxide basic (Acros Organics, Brockmann I, 50–200 µm, 60A), 2-(*n*-butylthiocarbonothioylthio)propanoic acid (BTPA, Boron Molecular, >95%), diphenyl (2,4,6-trimethylbenzoyl) phosphine oxide (TPO, Sigma-Aldrich, >97%), 2,2'-azobis(2-methylpropionitrile) solution (AIBN solution, Sigma-Aldrich, 0.2 M in toluene), acrylic acid (AA, anhydrous, Sigma-Aldrich, 99%) and poly(ethylene glycol) diacrylate average M_n = 250 (PEGDA, Sigma-Aldrich, >92%) were used as received. *n*-butyl acrylate (BA, Sigma-Aldrich, ≥99%) was passed through a basic aluminium oxide column to remove inhibitor prior to use.

Supplementary Methods

Nuclear magnetic resonance (NMR)

All NMR spectra were recorded on Bruker Avance III 400 MHz spectrometer using an external lock ($CDCl_3$).

Size exclusion chromatography (SEC)

Analysis of the molecular weight distributions of the polymers were determined using a Shimadzu modular system composed of an SIL-20A auto-injector, a Polymer Laboratories 5.0 µm bead-size guard column (50 × 7.5 mm^2) followed by three linear PL (Styragel) columns (10^5, 10^4 and 10^3), an RID-10A differential refractive-index (RI) detector, and a UV detector. The eluent was DMAc (containing 0.03% w/v LiBr and 0.05% w/v 2,6-dibutyl-4-methylphenol (BHT)) at 50 °C, run at a flow rate of 1.0 mL/min. The SEC was calibrated using narrow polystyrene (PSTY) standards with molecular weights of 200 – 10^6 g/mol.

Attenuated total reflectance - Fourier transform infrared (ATR-FTIR) spectroscopy

ATR-FTIR spectroscopy was performed to monitor photopolymerization kinetics using a Bruker Alpha FTIR spectrometer equipped with room temperature DTGS detectors. After taking a background reading of the empty plate, 20 µL of polymerization resin was pipetted onto the ATR crystal plate. An absorption spectrum was then obtained by scanning the droplet from 400-4000 cm^{-1}. After an initial reading, the droplet was irradiated with a Thorlabs mounted LED with a collimation adapter (λ_{max} = 405 nm, I_0 = 2.06 mW cm^{-2}) and subsequently the IR absorption spectra were obtained at various times to determine the integral of the vinylic peak at time t_x. Vinyl bond conversions were calculated from the disappearance of the C=C stretching peak at

1630 cm^{-1} normalized to the C=O stretching peak at 1760 cm^{-1} as an internal standard using Supplementary Equation (1):

$$Conversion\ (\%) = 100\ \times \left(1 - \frac{int_x/std_x}{int_0/std_0}\right) \quad (1)$$

Where int_x is the integral of the 1600-1650cm^{-1} peak at x min of irradiation, std_x is the integral of the 1670-1800 cm^{-1} peak at x min of irradiation, int_0 is the initial integral of the 1600-1650 cm^{-1} peak before irradiation, and std_0 is the initial integral of the 1670-1800 cm^{-1} peak before irradiation. The vinyl bond conversion was monitored using 15 s intervals between 0 to 2 min.

UV-Vis spectroscopy of resins

All UV-vis spectra of resins were recorded using a Varian Cary 300 spectrophotometer. Scans were conducted in the range of 600-200 nm at 600 nm/min. 2 mL of resins were placed in a 1×1 cm glass cuvette for measurements. A spectrum obtained with an empty cuvette was used for zero/baseline correction.

Fourier transform near-infrared (FTNIR) spectroscopy

FTNIR spectroscopy was performed using a Bruker Vertex 70 Fourier transform spectrometer. FTNIR spectroscopy was used to determine vinyl bond conversion of solid samples by comparing the integral of the of the C-H vinylic stretching overtone at 6120-6220 cm^{-1} between solid printed samples and a sample of uncured resin. Uncured resin (3 mL) was placed in a 1×1 cm quartz cuvette, and an absorption spectrum was obtained by scanning from 4000-8000 cm^{-1}. A solid 3D printed sample was measured using a digital caliper, placed in the cuvette and another absorption spectra was measured. The final conversion was calculated using Supplementary Equation (2):

$$Conversion\ (\%) = 100\ \times \left(1 - \left(\frac{int_f}{int_0} \times \frac{t_{cuvette}}{t_{sample}}\right)\right) \quad (2)$$

Where int_f is the integral of the peak from 6120-6220 cm^{-1} for the 3D printed sample, int_0 is the integral of the peak from 6120-6220 cm^{-1} for the unpolymerized resin, t_{sample} is the thickness of the sample, and $t_{cuvette}$ is the path length of the cuvette. Integrals were calculated using OPUS software 7.5.

Viscosity measurements of resins

All measurements were performed using ATAGO portable rotational viscometer under ambient conditions. Resin viscosity was measured at 200 rpm using an appropriate adapter (UL or SpindleA1)[1].

Atomic force microscopy (AFM)

All AFM measurements were performed on the Bruker Dimension ICON SPM, with a Nanoscope V controller (software version 9.70). An OTESPA-R3 probe (from Bruker AFM probes) was used to perform the tapping mode measurements. Mechanical properties measurements were performed using peak force tapping mode on

a top layer of printed object using the SCANASYST probe (from Bruker AFM probes). The scan size was set to 1 µm and 300 nm. The scan rate was set at around 0.6 to 0.7 Hz with a peakforce of approximately 500 pN. The feedback gain was adjusted accordingly to optimize tracking of the specimen surface, without any significant feedback noise. The resolution of the image was set to 512 pixels per line for 1 µm scan size and 256 samples/line for 300 nm scan size. For peakforce QNM measurements, the tip was calibrated using the thermal tunning method. AFM images were analysed using NanoScope Analysis software, version 1.7. For the statistical length analysis, at least 50 particles were carefully traced by hand to determine average domain size and domain spacing using ImageJ software. Histograms of the size distribution were constructed. Average PBA domain width (*D*), domain length (*L*) and domain spacing (*d*) were calculated using Supplementary Equation (3):

$$d_n = \frac{\sum_{i=1}^{n} N_i d_i}{\sum_{i=1}^{n} N_i} \tag{3}$$

Where *N* is the number of observations and *d* is the determined size for each measurement. *d* was defined as the centre-to-centre distance of the two PBA domains/phases. *D*, *L*, and *d* were defined according to Supplementary Fig. 10.

Small-angle X-ray scattering (SAXS)

SAXS experiments were performed on an Anton Paar SAXSPoint 2.0 system with a Cu K_α (λ = 0.154 nm) microfocus X-ray source (50 kV/1 mA) and Dectris Eiger 1M detector. Data was collected at room temperature, under vacuum for 5 min from a sample at a sample-to-detector distance of 0.575 m. Samples were 3D printed at the thickness of 2×100 µm layers. Data was reduced to 1D by radial averaging the 2D detector after converting pixel positions to $q = (4\pi/\lambda)\sin\theta$, where 2θ is the scattering angle). The domain spacing was calculated using Supplementary Equation (4):

$$d_{SAXS} = \frac{2\pi}{q} \tag{4}$$

Dynamic mechanical analysis (DMA)

DMA was performed using a single cantilever bending test. The sample dimensions were 40 mm (length) × 8 mm (width) × 2 mm (thickness). The analysis was performed using a TA instruments Q800 dynamic mechanical analyzer which was equipped with a TA instruments liquid nitrogen gas cooling accessory (GCA) for temperature control. Initially, the 3D printed sample was measured using digital calipers and placed into the single cantilever clamp. The clamp was then tightened using a torque wrench with a force of 5 in lb. All experiments were conducted using the following method: equilibration at -70 °C, isothermal for 3 min, temperature ramped to 150 °C at a rate of 2 °C/min, constant frequency of 1 Hz and displacement of 15 µm. Glass transition temperature was determined using the temperature at the peak of the tan δ curve.

SAXS fitting using Teubner-Strey (T-S) model

The position and the sharpness of SAXS peaks of microphase-separated 3D printed materials were fitted using T-S model[2] in SasView software. According to T-S model (Supplementary Equation (5))

$$I(q) = \frac{1}{a_2+c_1q^2+c_2q^4} + b \tag{5}$$

Where $q = (4\pi/\lambda)sin\theta$, λ is the wavelength, 2θ is the scattering angle; b is background scattering; a_2, c_1, c_2 are fitting parameters used to calculate domain spacing (d_{TS}), correlation length (ξ) and the amphiphilicity factor (f_a) using Supplementary Equations (6-8) below:

$$d_{TS} = 2\pi \left[\frac{1}{2}\left(\frac{a_2}{c_2}\right)^{1/2} - \frac{1}{4}\frac{c_1}{c_2}\right]^{-1/2} \tag{6}$$

$$\xi = \left[\frac{1}{2}\left(\frac{a_2}{c_2}\right)^{1/2} + \frac{1}{4}\frac{c_1}{c_2}\right]^{-1/2} \tag{7}$$

$$f_a = \frac{c_1}{\sqrt{4a_2c_2}} \tag{8}$$

Tensile testing

Dog-bone specimens were designed using Tinkercad 3D modelling software by modifying ASTM D638 Type I specimen[3] and the object was exported as an .stl file. Specimen dimensions were thickness (T) = 2.04 mm, width overall (WO) = 8.38 mm, length overall (LO) = 50.3 mm, distance between grips (D) = 36 mm, gauge length (G) = 15.79 mm, width at the centre = 6 mm.

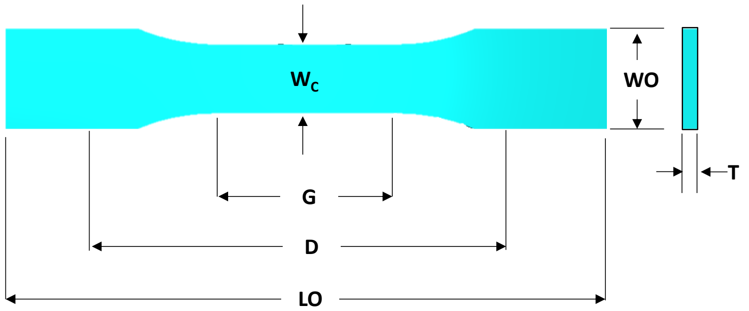

The mechanical tensile stress tests were performed using a Mark–10 ESM303 with a 1 kN force gauge model M5–200. The speed of testing was 1.1 mm/min. All tensile results were performed in at least triplicate. The tensile stress was calculated from the applied force divided by the initial cross-sectional area of the gage section. The strain was determined as the change in gage length relative to the original specimen gage length, expressed as a percent. Toughness was determined by calculating the area under a stress-strain curve using the trapezoidal rule.

Compression testing

Materials for compression testing were designed based on the work of Gu and coworkers[10]. A 30th-order Bezier curve with 31 control points was generated from x-y coordinates, based on a target density of 0.1734 and using a lattice constant of 0.9 (to produce a 0.9 cm^3 unit cell). The Bezier curve was rotated 360° about its long axis

to provide a single strut. The struts were arranged to form a body cubic centred unit cell, which was further replicated to provide a lattice with target dimensions 27 × 27 × 27 mm^3. Compression testing of the 3D printed lattice structures was performed using a universal testing machine (Instron 3369, 50 kN). The specimen was compressed in the Z-direction at a rate of 1 mm min^{-1}. All compression results were performed in triplicate.

Scanning electron microscopy (SEM)

The fractography of selected failed tensile samples was studied via SEM. Samples were set on a double 90° angled stub using a conductive tape and coated with platinum coating with 15 nm thickness using a Leica ACE600 sputter coater. Hitachi TM4000Plus was used for imaging. An accelerating voltage of 15 kV, standard vacuum, and backscattered electron (BSE) detector were applied during imaging.

Estimation of $\chi_{\text{P(AA-stat-PEGDA)-b-PBA}}$ by group molar contribution method

Supplementary Note 1: $\chi_{\text{P(AA-stat-PEGDA)-b-PBA}}$ was estimated using Supplementary Equation (9):

$$\chi_{\text{P(AA}-stat-\text{PEGDA)}-b-\text{PBA}} = (1-x)\chi_{\text{PEGDA-PBA}} + x\chi_{\text{PAA-PBA}} + x(1-x)\chi_{\text{PAA-PEGDA}} \quad (9)$$

where x is the weight fraction of AA in P(AA-*stat*-PEGDA) block (x = 0.54). χ_{12} was calculated using Supplementary Equation (10):

$$\chi_{12} = \frac{VN_A}{RT}(\delta_1 - \delta_2)^2 \quad (10)$$

where V is the reference volume (set to 118 $Å^3$), R is the gas constant (1.987 cal mol^{-1} K^{-1}), T is temperature (set to 298 K), N_A is the Avogadro's number (6.02 × 10^{23} mol^{-1}), δ ((cal $cm^{-3})^{1/2}$) is solubility parameter estimated using the group molar contribution method proposed by Small[4] (Supplementary Equation (11)):

$$\delta = \frac{d\Sigma G}{M} \quad (11)$$

where d (g cm^{-3}) is density, M is monomer molecular weight, $\sum G$ is the sum of the molar attraction constants. Estimated δ values were as follows: δ_{PBA} = 9.15 $cal^{1/2}$ $cm^{-3/2}$, δ_{PAA} = 11.18 $cal^{1/2}$ $cm^{-3/2}$, δ_{PEGDA} = 8.50 $cal^{1/2}$ $cm^{-3/2}$. Then, χ parameters were calculated using Supplementary Equation (10): $\chi_{\text{PEGDA-PBA}}$ = 0.051, $\chi_{\text{PAA-PBA}}$ = 0.497, $\chi_{\text{PAA-PEGDA}}$ = 0.865. Subsequently, $\chi_{\text{P(AA-}stat\text{-PEGDA)-}b\text{-PBA}}$ was calculated using Supplementary Equation (9): $\chi_{\text{P(AA-}stat\text{-PEGDA)-}b\text{-PBA}}$ = 0.505.

Synthetic Procedures

RAFT polymerization of *n*-butyl acrylate in acetonitrile

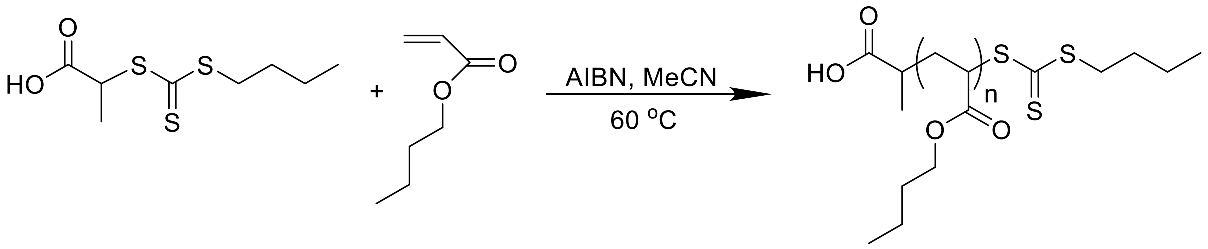

Supplementary Fig. 1. Synthesis of PBA_n-CTA using RAFT polymerization of *n*-butyl acrylate.

Protocol for the synthesis of PBA_{24}-CTA: *n*-butyl acrylate (70 g, 0.546 mol), BTPA RAFT agent (5.21 g, 2.2×10^{-2} mol) and AIBN (0.2 M solution in toluene, 16.4 mL, 3.3×10^{-3} mol) were dissolved in acetonitrile (125 mL). The mixture was deoxygenated by purging with nitrogen for 90 min, and then polymerized for 15 h at 60 °C. The reaction was stopped by cooling inside a freezer (-20 °C) for 30 min and exposing to air. The polymer solution was concentrated by rotary evaporation and used without further purification. Using the same protocol, other PBA-CTAs were synthesized. The characterizations for PBA-CTAs are summarized in Supplementary Table 1.

Supplementary Table 1. Characterization of PBA-CTAs synthesized by RAFT-mediated polymerization of *n*-butyl acrylate in acetonitrile.

MacroCTA	Conversion (%)[a]	M_n (theory) (kg/mol)[b]	SEC (RI, DMAc)[c]		^{1}H NMR[d]		
			M_n (kg/mol)	$Đ$	X_n	M_n (kg/mol)	End-group fidelity, f (%)
PBA_{24}-CTA	96	3.3	3.4	1.06	24	3.3	92
PBA_{48}-CTA	97	6.4	6.0	1.11	48	6.4	93
PBA_{94}-CTA	94	12.3	9.6	1.11	94	12.3	91
PBA_{180}-CTA	90	23.3	20.2	1.09	180	23.3	90
PBA_{360}-CTA	90	46.4	38.9	1.19	360	46.4	85

[a] Monomer conversion was calculated by ^{1}H NMR by comparing integrals of polymers (4.05 ppm) and residual monomers (~ 6 ppm). [b] M_n (theory) = ([BA]/[BTPA]) × conv. (BA) × MW(BA) + MW(BTPA). [c] DMAc (containing 0.03% w/v LiBr and 0.05% w/v 2,6-dibutyl-4-methylphenol (BHT)) as eluent with polystyrene as calibration standards. [d] ^{1}H NMR (400 MHz, $CDCl_3$) at 298 K. The degree of polymerization (X_n) of PBA-CTAs was calculated based on the integral value at 4.05 ppm (the peak a in Supplementary Fig. 2), $X_n = I_{4.05}/2$. M_n (NMR) = X_n(PBA-CTA) × MW(BA) + MW(BTPA). End group fidelity was calculated using Supplementary Equation (12):

$$f\ (\%) = \frac{I_{4.8}}{\left(\frac{I_{3.35}}{2}\right)} \times 100\% \qquad (12)$$

where $I_{3.35}$ and $I_{4.8}$ are integral values at 3.35 and 4.8 ppm, respectively, which represent the peaks c and d in Supplementary Fig. 3.

Additional Data

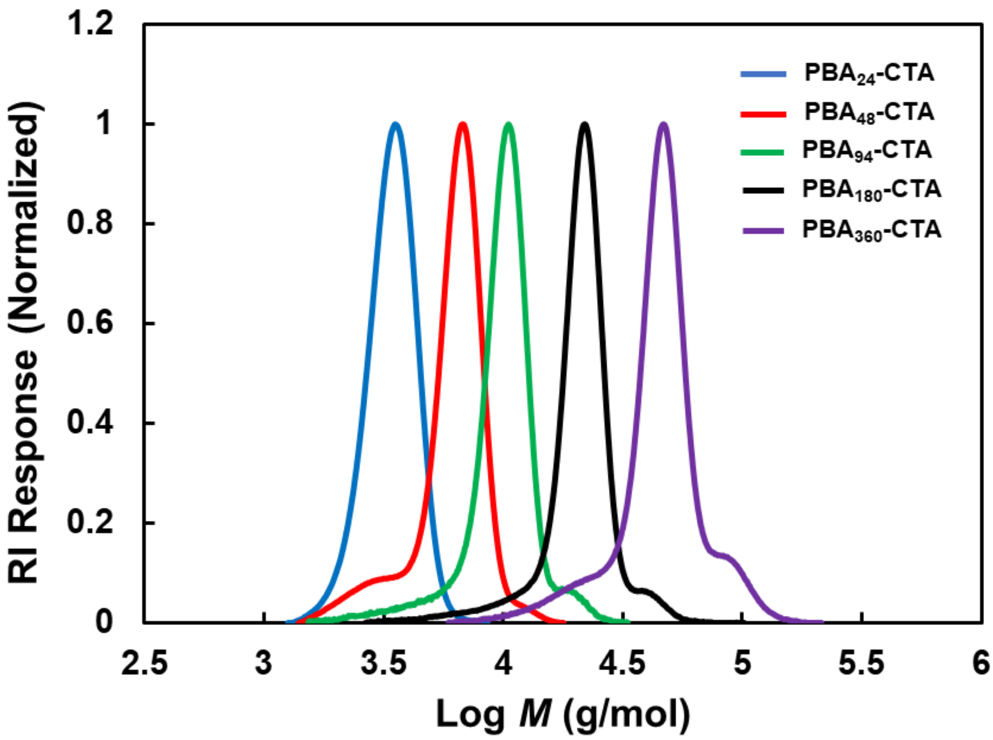

Supplementary Fig. 2. Molecular weight distributions of PBA_n-CTAs obtained by SEC using a RI detector, with DMAc as eluent (containing 0.03% w/v LiBr and 0.05% w/v 2,6-dibutyl-4-methylphenol (BHT)) and calibrated using PSTY standards.

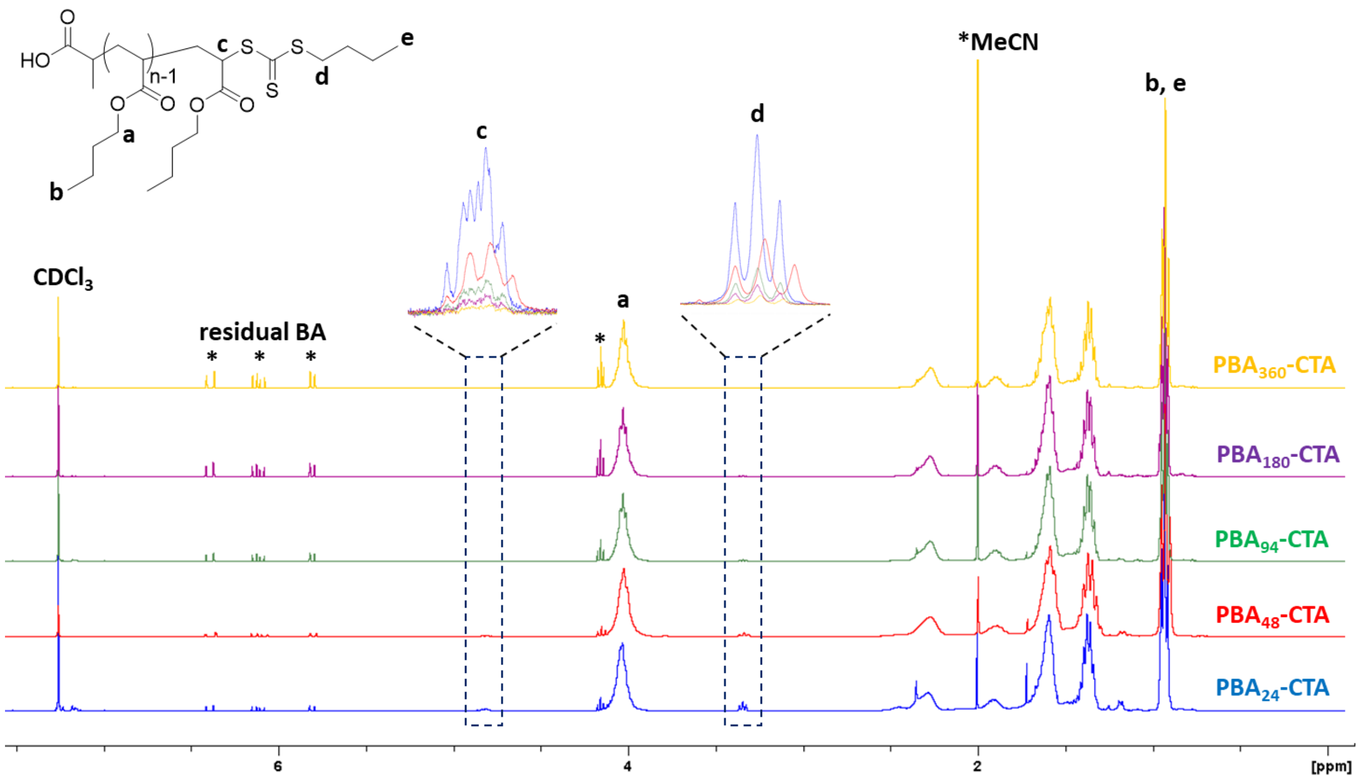

Supplementary Fig. 3. ^{1}H NMR spectra (400 MHz, $CDCl_3$, 298 K) of PBA_n-CTAs. * - residual BA monomer and residual acetonitrile (MeCN). The spectra normalized by a resonance at 4.03 ppm.

Supplementary Table 2. Resin formulations with varying degree of polymerization (X_n) of PBA-CTA and corresponding conversions of 3D printed objects. A molar ratio of [AA]/[PEGDA] was fixed at 4/1.

Resin #	X_n of PBA-CTA[a]	Resin components (wt%)				Resin conversion (%)[b]
		PBA_n-CTA	AA	PEGDA	TPO	
**1**	24					93
**2**	48					94
**3**	94	16.5	44.5	38.7	0.3	91
**4**	180					94
**5**	360					92
**6**	24					96
**7**	48					95
**8**	94	28.2	38.2	33.1	0.5	93
**9**	180					96
**10**	360					95
**11**	24					97
**12**	48					97
**13**	94	43.9	29.7	25.7	0.7	95
**14**	180					96
**15**	360					96
**16**	360	16.5	44.7	38.7	0.133	93

[a] – the degree of polymerization of PBA-CTA determined by ^{1}H NMR. [b] – Conversions measured by comparing printed samples to uncured resins using FTNIR analysis.

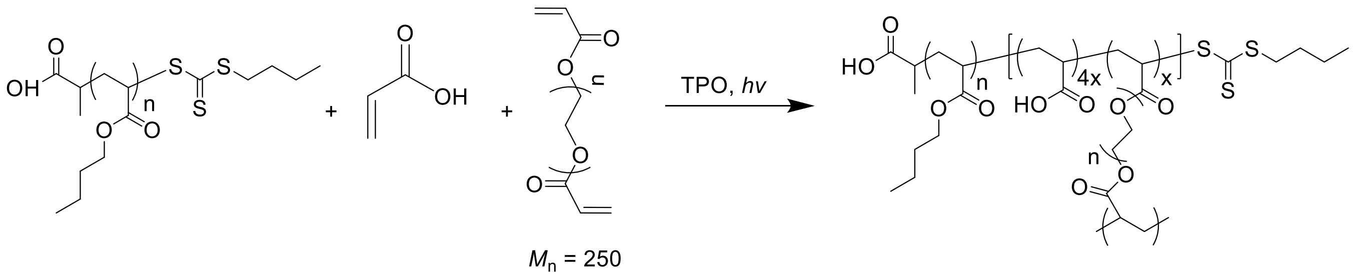

Supplementary Fig. 4. Photoinitiated RAFT copolymerization of AA and PEGDA in the presence of PBA_n-CTA and TPO, as performed in the DLP 3D printer.

Supplementary Table 3. Viscosity for the indicated resins measured at 200 rpm.

Resin #	wt% loading of PBA-CTA	X_n of PBA-CTA	Viscosity (mPa s)
**1**	16.5	24	9.97
**2**		48	12.50
**3**		94	16.95
**4**		180	23.34
**5**		360	40.65[a]
**1**	16.5	24	9.97
**6**	28.2		17.65
**11**	43.9		46.41
**5**	16.5	360	40.65[a]
**10**	28.2		123.6
**15**	43.9		494.1[b]

[a] – This viscosity value is reported at 100 rpm (viscosity reached plateau). [b] – This viscosity value is reported at 150 rpm (viscosity value reached plateau and cannot be measured at higher rpm due to torque is being outside of recommended measurement range (10 – 100%) of prescribed torque).

Supplementary Note 2: The viscosities of the selected resins were determined to investigate the effects of PBA-CTA X_n and PBA-CTA wt%. At fixed wt% of PBA-CTA (16.5 wt%), the viscosities of resins increase from 9.97 mPa s for PBA_{24}-CTA to 40.65 mPa s for PBA_{360}-CTA. At fixed PBA-CTA X_n (X_n = 24), the viscosities of resins increase from 9.97 mPa s at 16.5 wt% loading to 17.65 mPa s at 28.2 wt% loading and further to 46.41 mPa s at 43.9 wt% loading. The similar trend was observed for the viscosities of resins consisting of PBA_{360}-CTA as a function of wt% (40.65 mPa s at 16.5 wt%, 123.6 mPa s at 28.2 wt% and 494.1 mPa s at 43.9 wt%). It should be noted that all resins were suitable for DLP 3D printing.

Supplementary Table 4. Molar ratios of [AA]/[PEGDA]/[PBA_n-CTA] for various resin formulations at three wt% loading of PBA-CTA (16.5, 28.2 and 43.9 wt%). A molar ratio of [AA]/[PEGDA] was fixed at 4/1.

**Resin #**	**wt% loading of PBA-CTA**	**X_n of PBA-CTA[a]**	**Molar ratio between resin components**			**Number of propagating chains (number L^{-1})[b]**	**Resin conversion (%)[c]**	**N[d]**	**χN[e]**
			**AA**	**PEGDA**	**PBA_n-CTA**				
**1**	16.5	24	124	31	1	3.24×10^{22}	93	168	85
**2**		48	240	60	1	1.68×10^{22}	94	330	167
**3**		94	461	115	1	8.73×10^{21}	91	618	312
**4**		180	875	219	1	4.60×10^{21}	94	1208	610
**5**		360	1742	435	1	2.31×10^{21}	92	2363	1193
**6**	28.2	24	62	16	1	5.55×10^{22}	96	99	50
**7**		48	120	30	1	2.88×10^{22}	95	191	96
**8**		94	231	58	1	1.50×10^{22}	93	363	183
**9**		180	438	109	1	7.90×10^{21}	96	705	356
**10**		360	871	218	1	3.97×10^{21}	95	1395	704
**11**	43.9	24	31	8	1	8.66×10^{22}	97	62	31
**12**		48	60	15	1	4.49×10^{22}	97	121	61
**13**		94	115	29	1	2.34×10^{22}	95	231	116
**14**		180	219	55	1	1.23×10^{22}	96	443	224
**15**		360	435	109	1	6.19×10^{21}	96	882	445

[a] – The degree of polymerization (X_n) of PBA-CTA determined by ^{1}H NMR. [b] – The number of propagating chains was estimated using Supplementary Equation (13):

$$number\ of\ chains\ per\ liter = \frac{m}{M_n \times V} \times N_A \qquad (13)$$

Where m (g) – mass of PBA-CTA in a resin formulation; M_n (g/mol) –number-average molecular weight of PBA-CTA determined by ^{1}H NMR; V – total volume of resin (L); N_A – Avogadro number (6.02×10^{23} mol^{-1}). [c] – Resin conversion was measured by comparing printed samples to uncured resins using FTNIR analysis.

[d] – $N = N_{PBA} + N_{net\text{-}P(AA\text{-}stat\text{-}PEGDA)}$.

[e] – Estimated $\chi = 0.505$. See details in Supplementary Note 1.

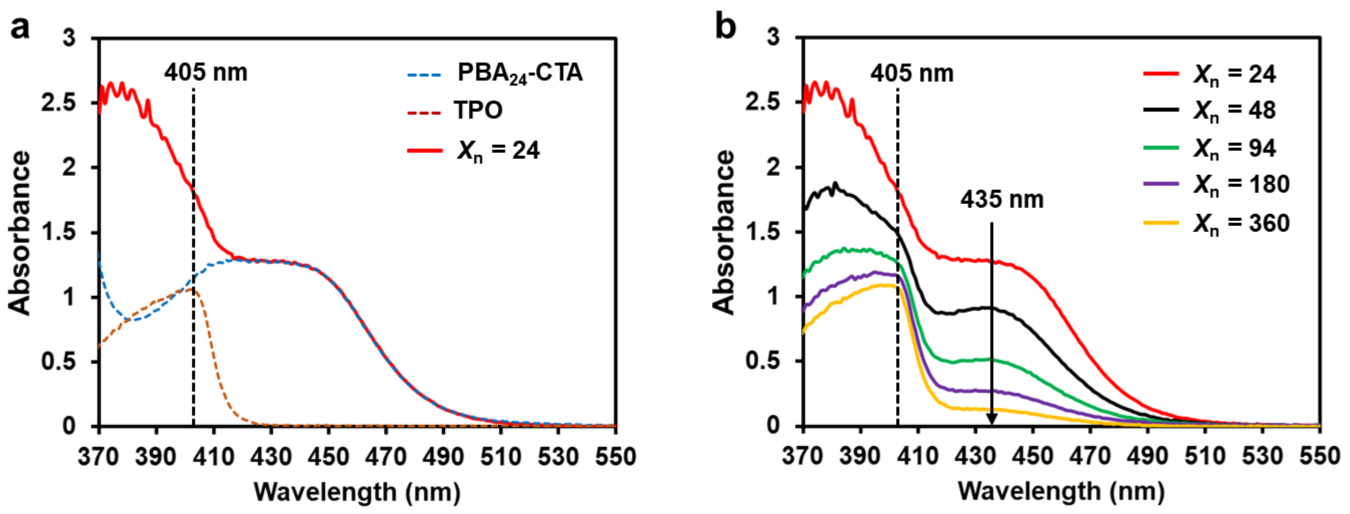

Supplementary Fig. 5. UV-Vis spectra of PBA-CTA and TPO mixtures. **a** UV-Vis spectra of PBA_{24}-CTA, TPO and a resin containing 16.5 wt% of PBA_{24}-CTA and 0.3 wt% of TPO. **b** UV-Vis spectra of resins formulated with 16.5 wt% of PBA-CTA with various degree of polymerization (X_n) and 0.3 wt% of TPO. The resins were formulated at a fixed molar ratio of [AA]/[PEGDA] = 4/1.

Supplementary Note 3: It can be seen from Supplementary Fig. 5a that UV-Vis absorbance spectra of TPO and PBA-CTA overlap in the region of 370 – 430 nm, indicating competitive light absorption at 405 nm (the operating wavelength of Anycubic Photon S 3D printer). Upon increasing X_n of PBA-CTA from 24 to 360 repeating units and at a fixed molar ratio of [AA]/[PEGDA] = 4/1 and fixed wt% of PBA-CTA in a resin, the concentration of RAFT containing polymer chains decreases. This confirmed by the decrease in the absorbance at 435 nm, which corresponds to the spin forbidden $n \rightarrow \pi^*$ transition of the thiocarbonyl group (Supplementary Fig. 5b).

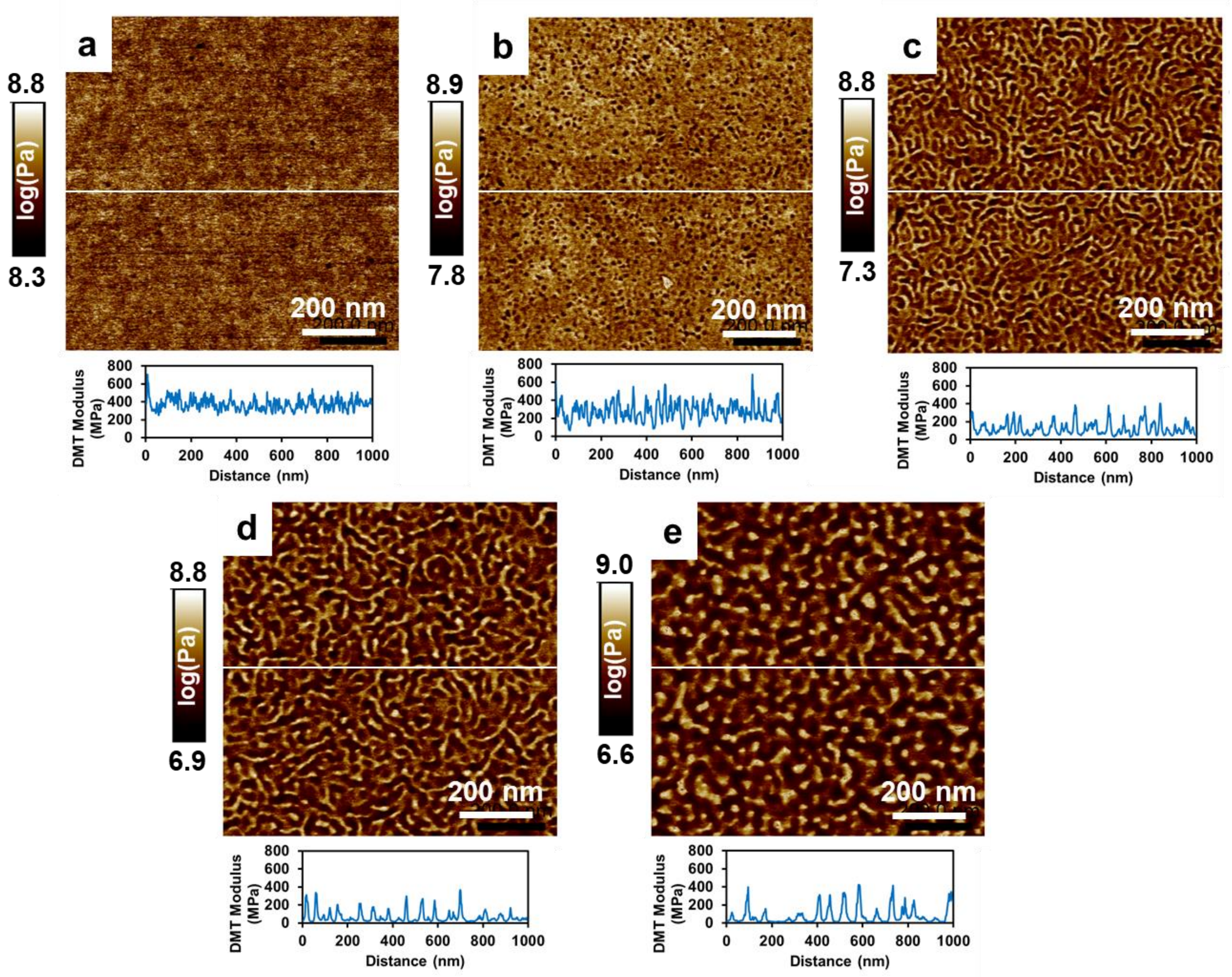

Supplementary Fig. 6. PeakForce QNM modulus map images and line-cut analysis of samples 3D printed using PBA-CTAs with various degree of polymerization (X_n): **a** X_n = 24; **b** X_n = 48; **c** X_n = 94; **d** X_n = 180; **e** X_n = 360. Materials were 3D printed using a molar ratio of [AA]/[PEGDA] = 4/1 at 16.5 wt% loading of PBA-CTA.

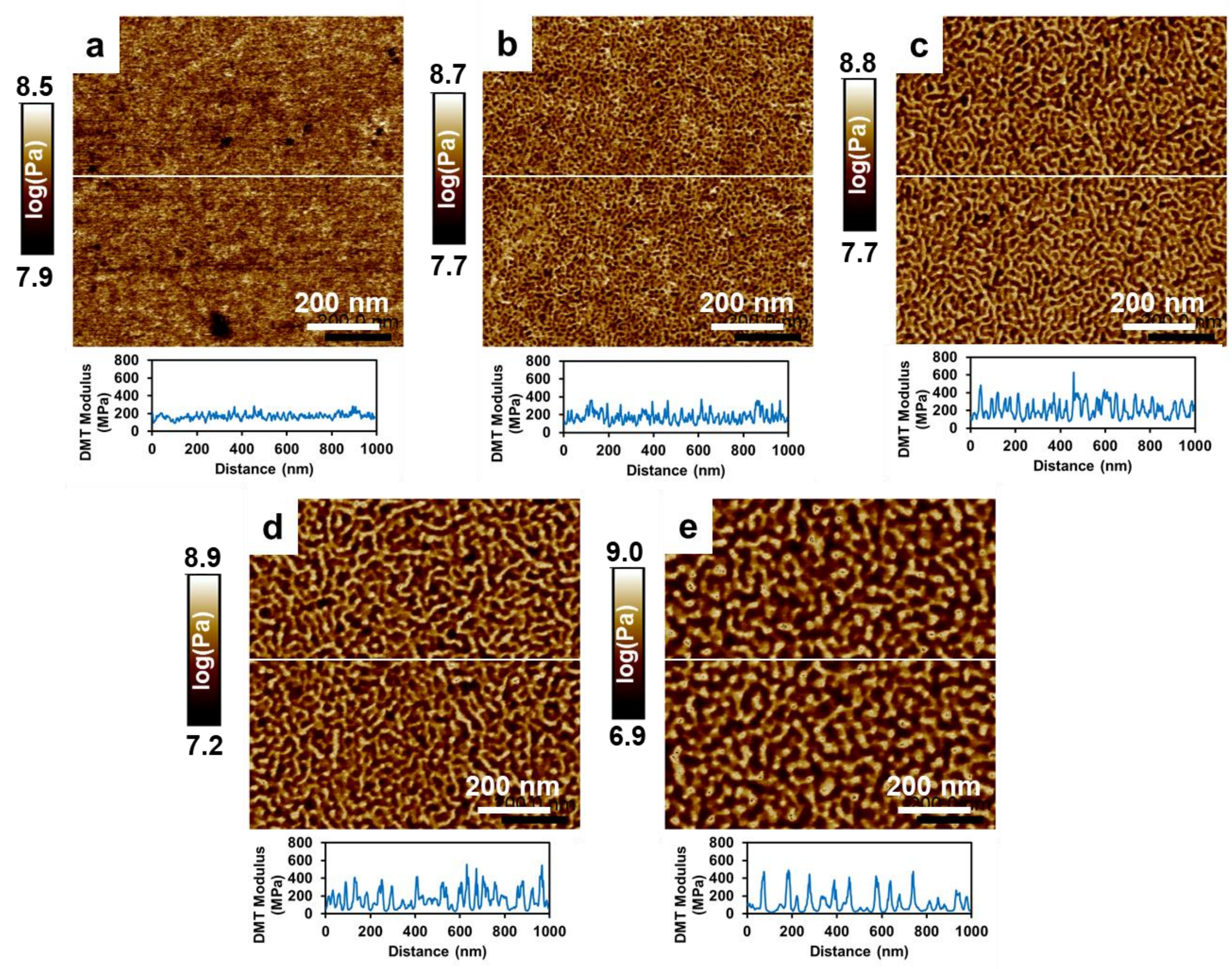

Supplementary Fig. 7. PeakForce QNM modulus map images and line-cut analysis of samples 3D printed using PBA-CTAs with various degree of polymerization (X_n): **a** X_n = 24; **b** X_n = 48; **c** X_n = 94; **d** X_n = 180; **e** X_n = 360. Materials were 3D printed using a molar ratio of [AA]/[PEGDA] = 4/1 at 28.2 wt% loading of PBA-CTA.

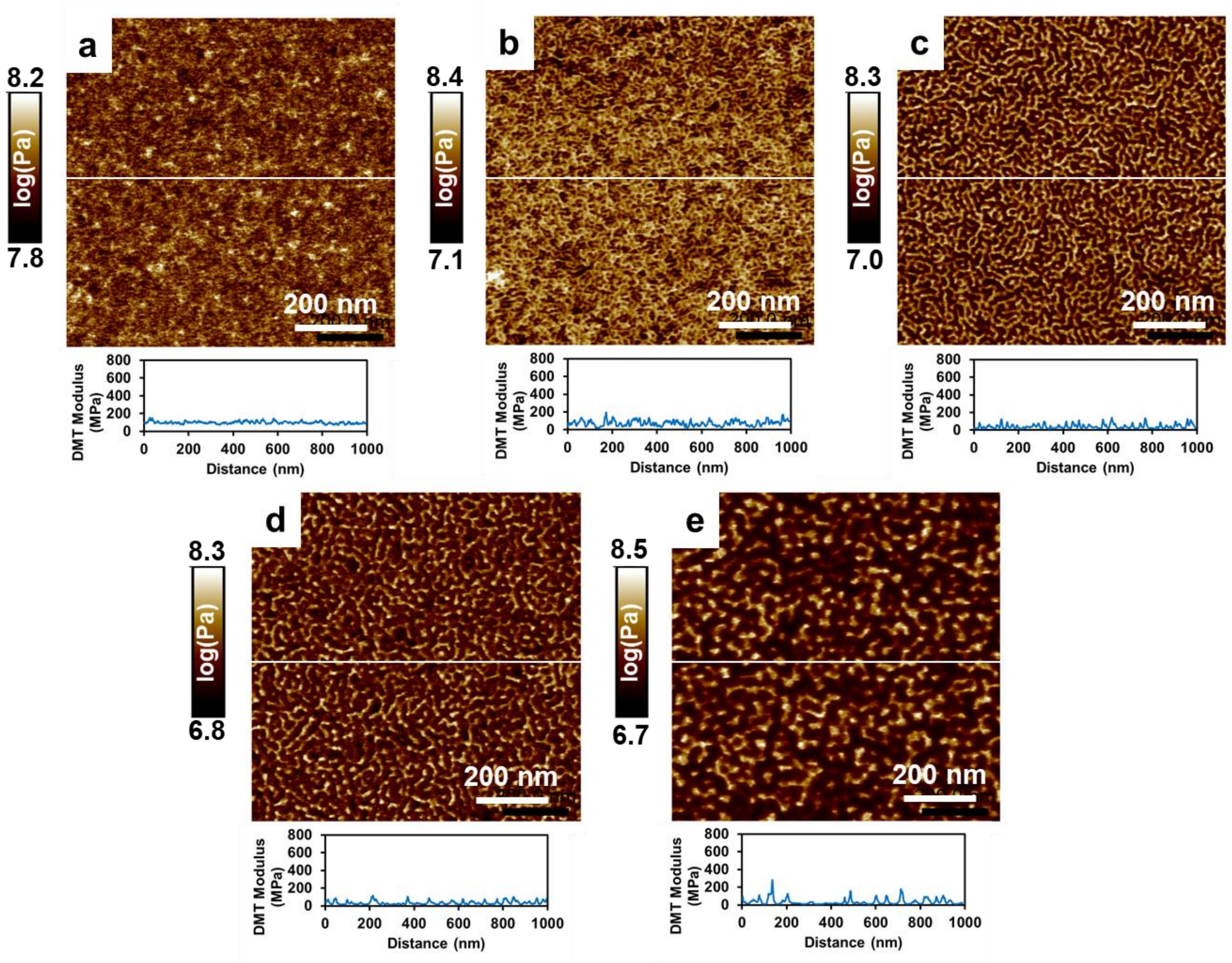

Supplementary Fig. 8. PeakForce QNM modulus map images and line-cut analysis of samples 3D printed using PBA-CTAs with various degree of polymerization (X_n): **a** X_n = 24; **b** X_n = 48; **c** X_n = 94; **d** X_n = 180; **e** X_n = 360. Materials were 3D printed using a molar ratio of [AA]/[PEGDA] = 4/1 at 43.9 wt% loading of PBA-CTA.

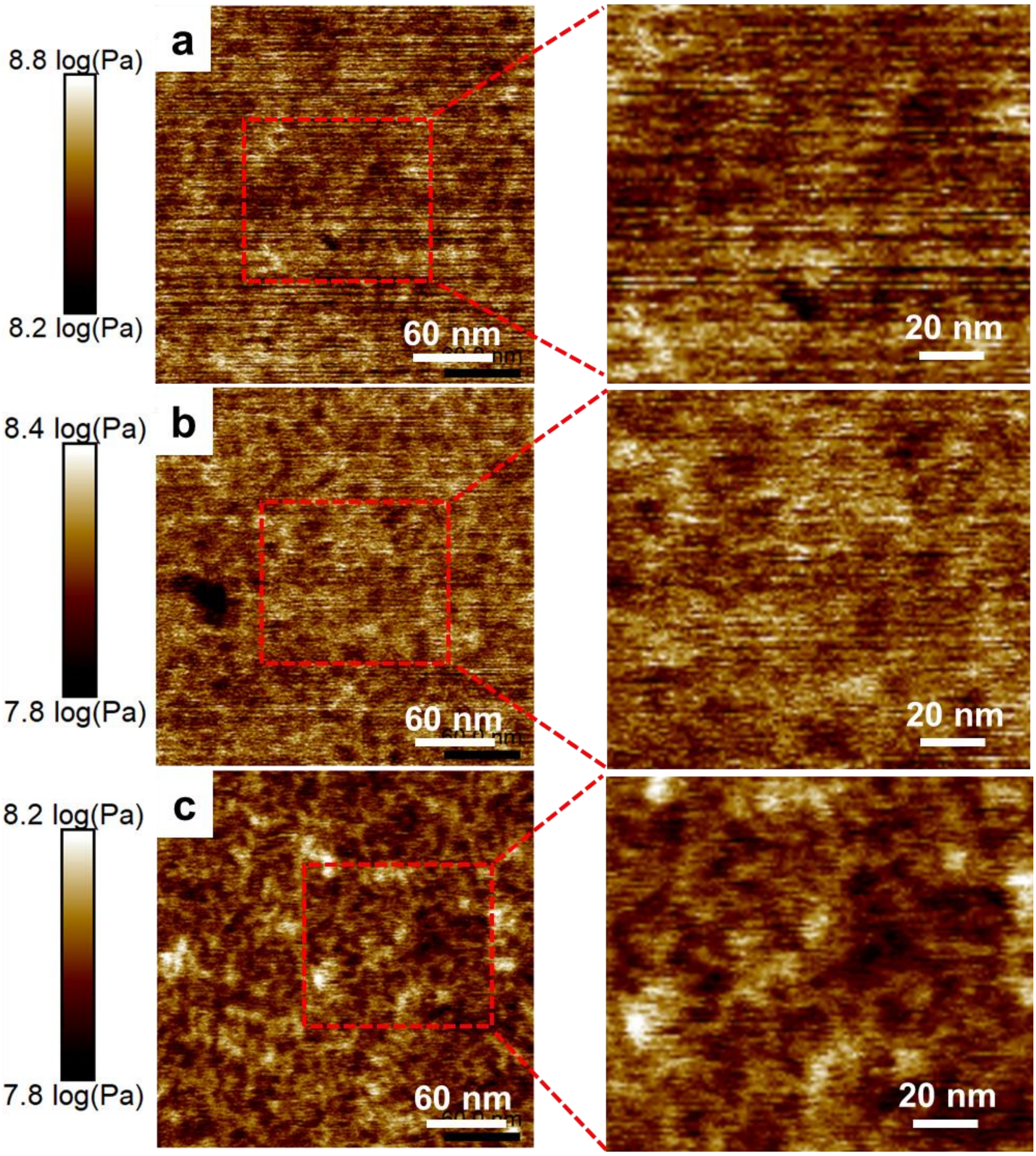

Supplementary Fig. 9. PeakForce QNM modulus map images of samples 3D printed using PBA_{24}-CTA at three different weight percentage of PBA-CTA in a resin formulation: **a** 16.5 wt.%; **b** 28.2 wt%; **c** 43.9 wt%. Materials were 3D printed using a molar ratio of [AA]/[PEGDA] = 4/1.

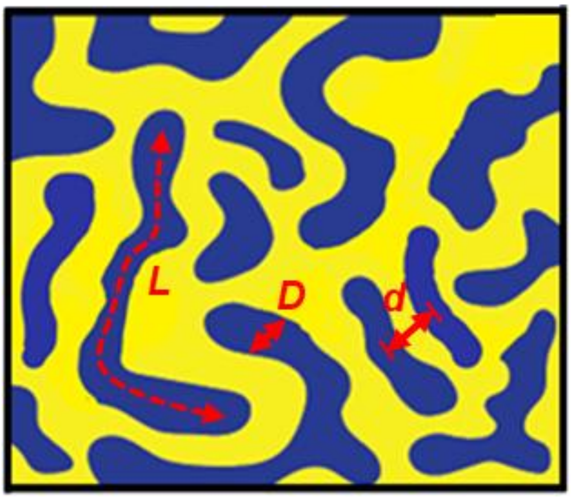

Supplementary Fig. 10. Schematic illustration of microphase-separated morphology. PBA domains are shown in blue; *net*-P(AA-*stat*-PEGDA) domains are shown in yellow. *D* – PBA domain width (D_{PBA}); *L* – PBA domain length; *d* – domain spacing.

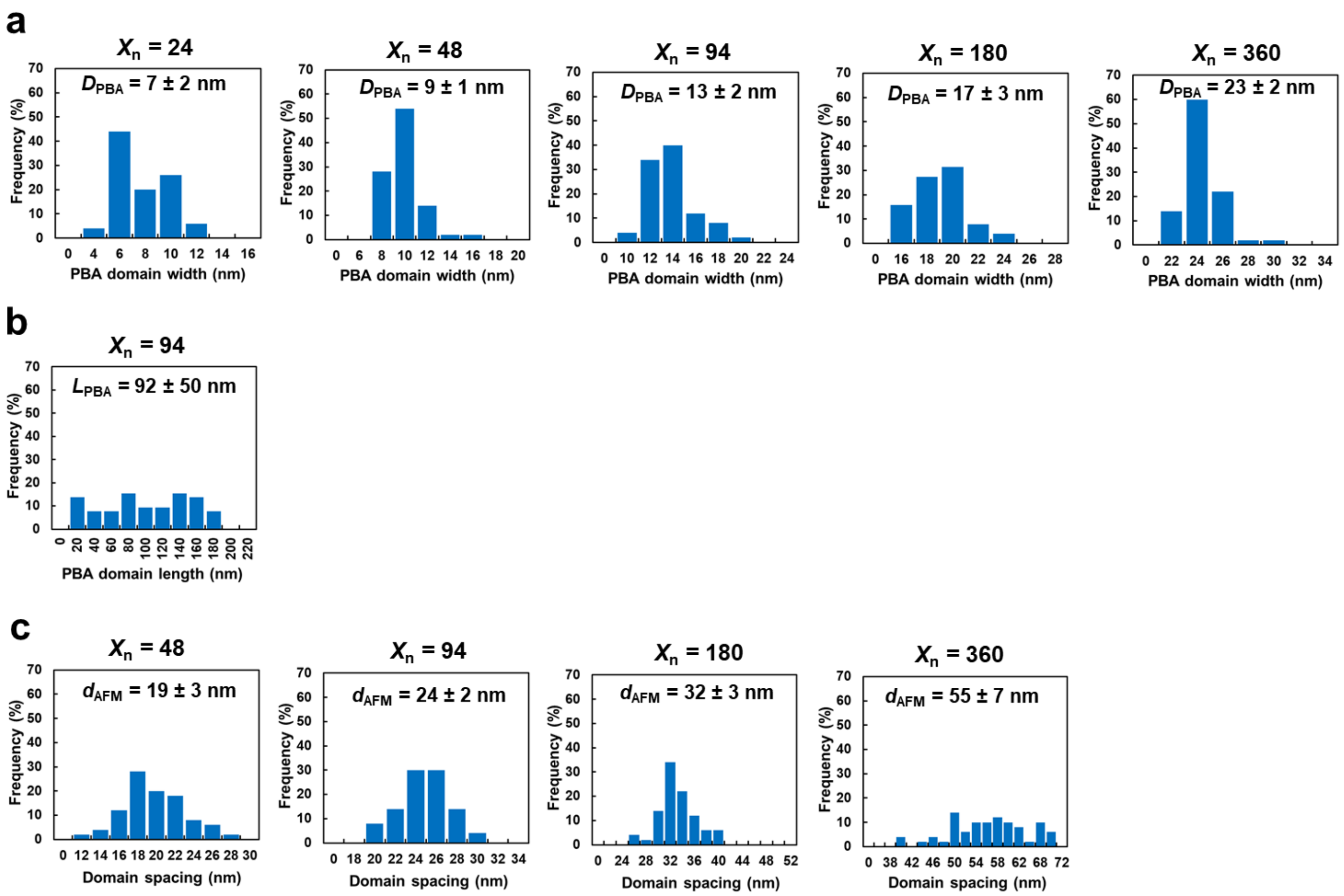

Supplementary Fig. 11. Average PBA domain width and domain spacing for materials 3D printed using PBA-CTAs with various degree of polymerization (X_n). **a** Domain width (D_{PBA}); **b** Domain length (L_{PBA}); **c** Domain spacing (d_{AFM}). Materials were 3D printed using a molar ratio of [AA]/[PEGDA] = 4/1 at 16.5 wt% loading of PBA-CTA. d_{AFM} for X_n = 24 was not reported due to the difficulty in precise measurements.

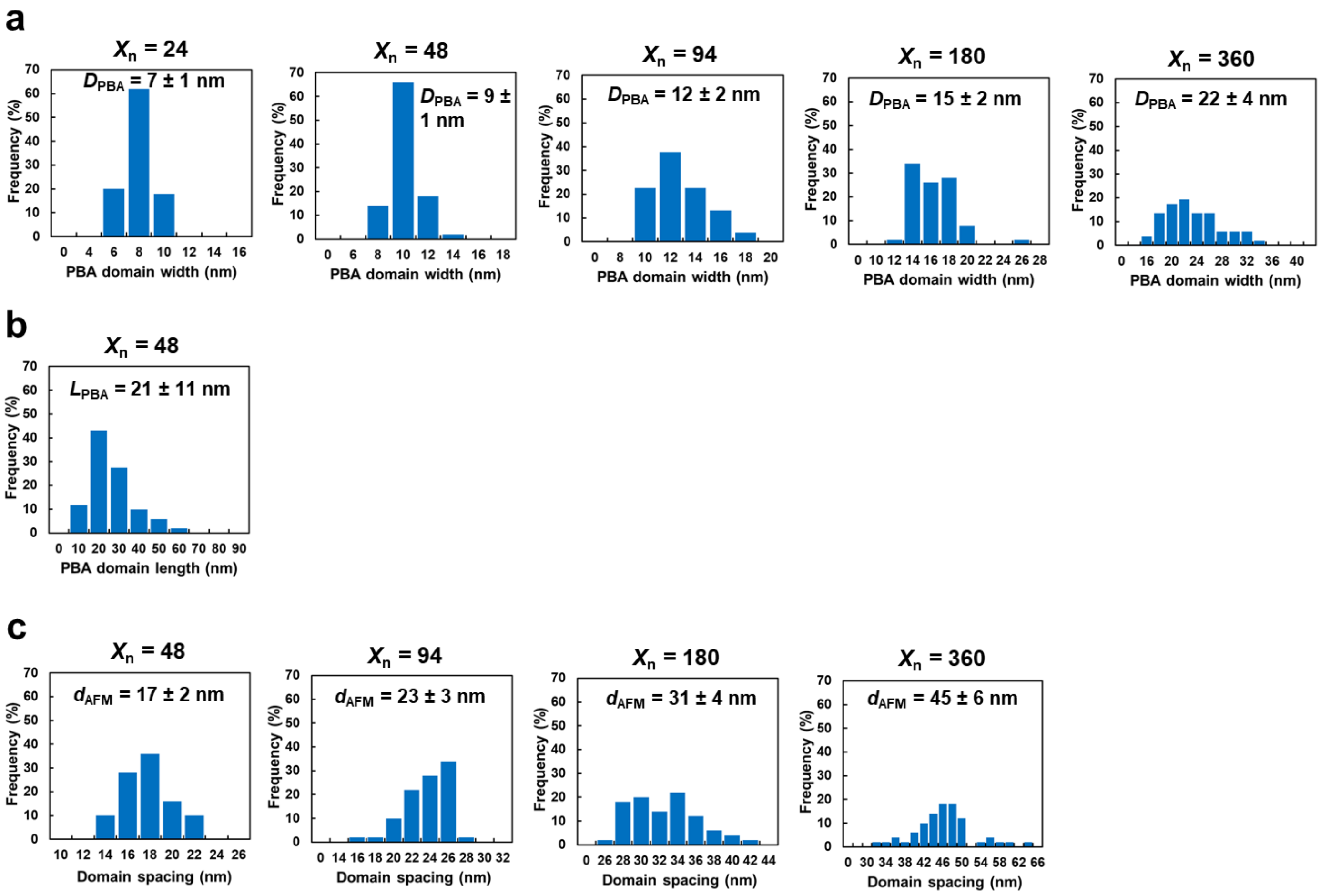

Supplementary Fig. 12. Average PBA domain width and domain spacing for materials 3D printed using PBA-CTAs with various degree of polymerization (X_n). **a** Domain width (D_{PBA}); **b** Domain length (L_{PBA}); **c** Domain spacing (d_{AFM}). Materials were 3D printed using a molar ratio of [AA]/[PEGDA] = 4/1 at 28.2 wt% loading of PBA-CTA. d_{AFM} for X_n = 24 was not reported due to the difficulty in precise measurements.

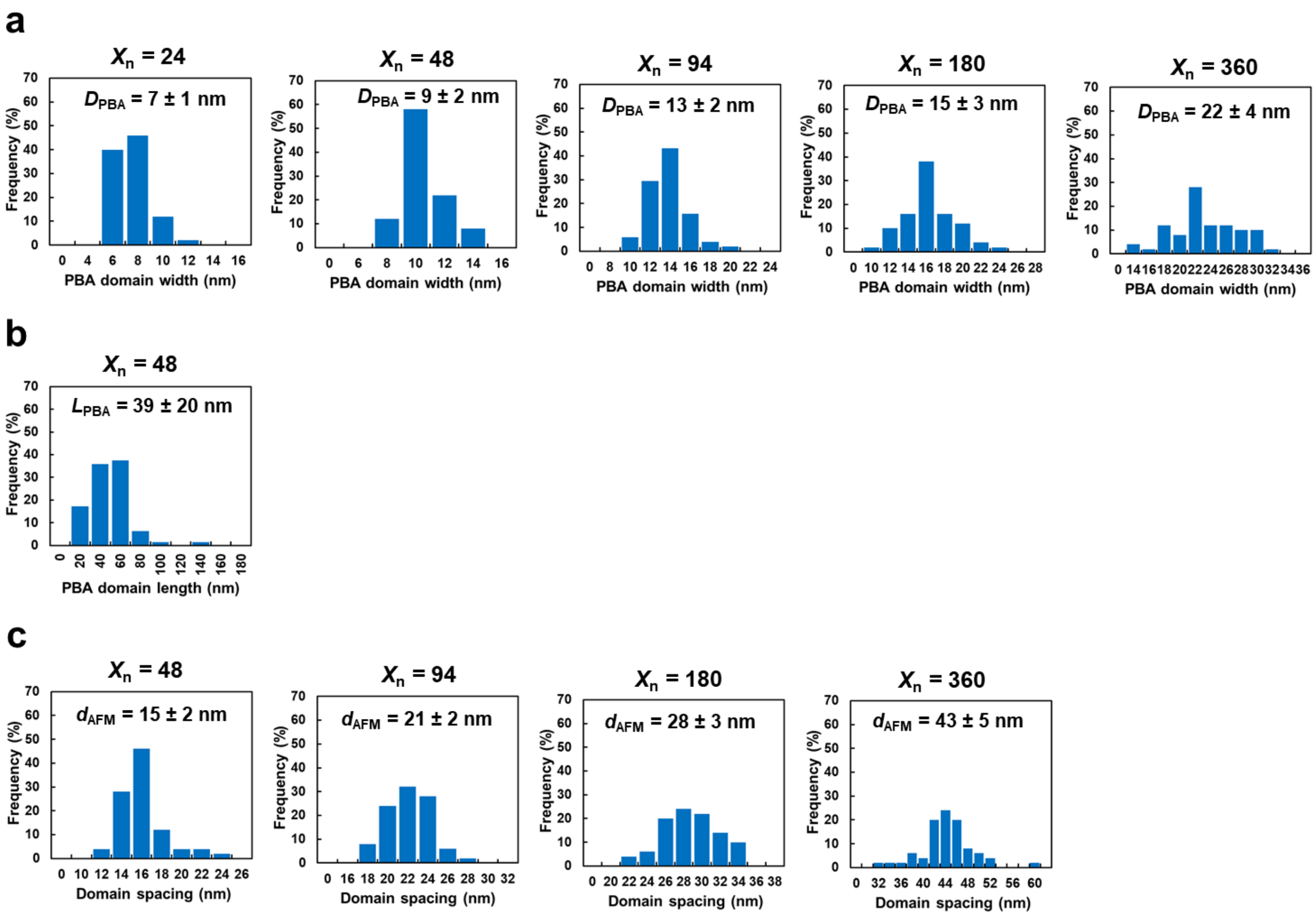

Supplementary Fig. 13. Average PBA domain width and domain spacing for materials 3D printed using PBA-CTAs with various degree of polymerization (X_n). **a** Domain width (D_{PBA}); **b** Domain length (L_{PBA}); **c** Domain spacing (d_{AFM}). Materials were 3D printed using a molar ratio of [AA]/[PEGDA] = 4/1 at 43.9 wt% loading of PBA-CTA. d_{AFM} for X_n = 24 was not reported due to the difficulty in precise measurements.

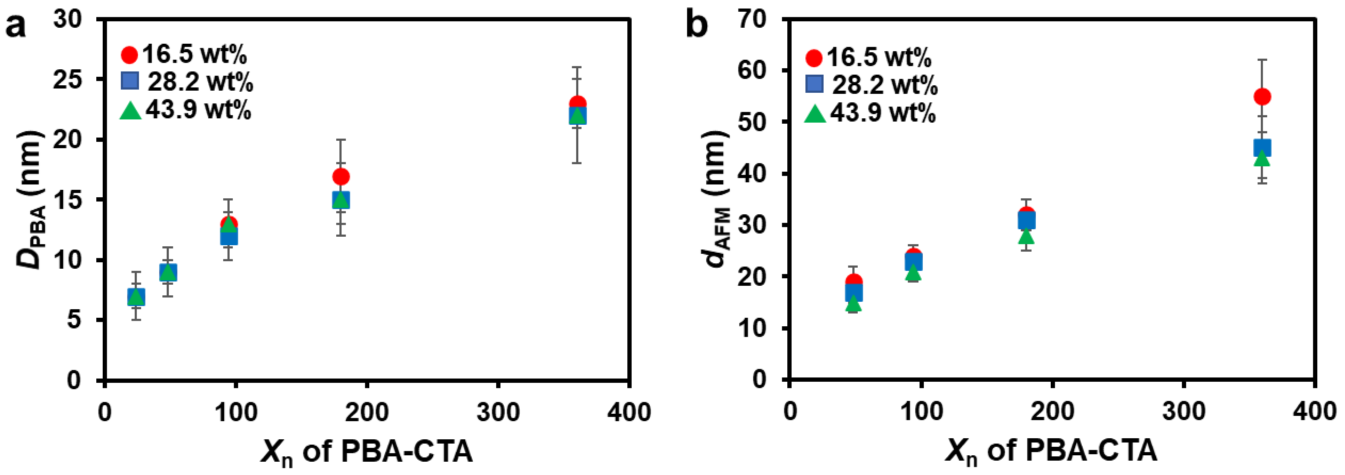

Supplementary Fig. 14. Dependence of **a** PBA domain width (D_{PBA}) and **b** domain spacing (d_{AFM}) for materials 3D printed using PBA-CTAs with various degree of polymerization (X_n) and mass loading (16.5, 28.2 and 43.9 wt%). D_{PBA} and d_{AFM} values were measured manually by analysing AFM images using ImageJ software. Error bars represent the standard deviation of at least 50 measurements.

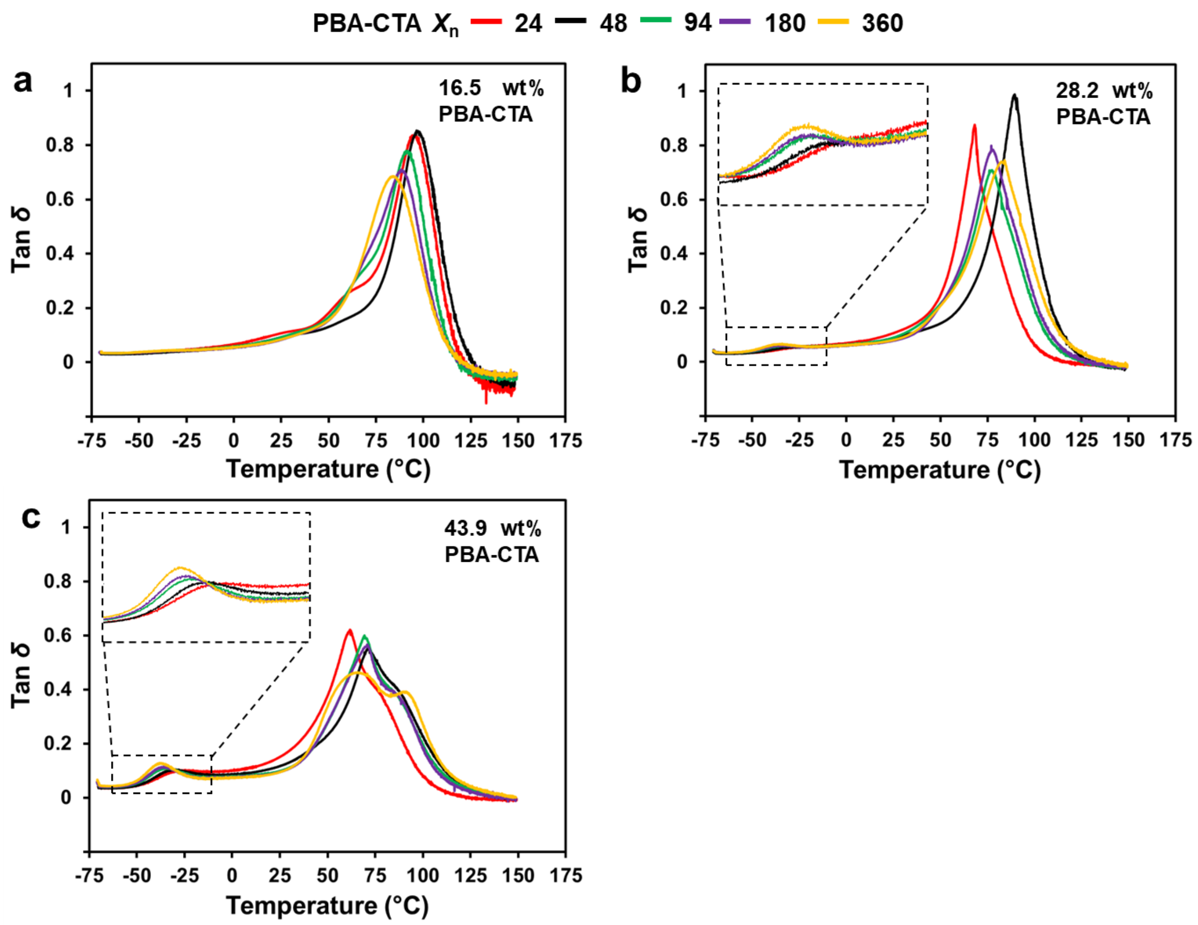

Supplementary Fig. 15. Tan δ profiles for materials 3D printed using resins with various degree of polymerization (X_n) of PBA-CTA at three different weight percentage of PBA-CTA in a resin formulation: **a** 16.5 wt.%; **b** 28.2 wt%; **c** 43.9 wt%. Materials were 3D printed using a molar ratio of [AA]/[PEGDA] = 4/1.

Supplementary Note 4: Tan δ profiles of materials 3D printed with the lowest loading of PBA-CTA (16.5 wt%) showed only one broad peak at ~ 90 ºC associated with *net*-P(AA-*stat*-PEGDA) phase.[5] The peak position shifted from 98 to 85 °C upon increasing X_n of PBA block. The peak attributed to PBA phase was not observed clearly, which is likely attributed to low weight loading of macroCTA. Tan δ profiles of materials 3D printed with 28.2 and 43.9 wt% of PBA-CTA in the resin demonstrated two distinct peaks: the higher temperature broad tan δ peak is attributed to the *net*-P(AA-*stat*-PEGDA) phase[5] and the lower temperature tan δ peak was seen around -35 °C and associated with the PBA-rich phase[6]. The lower temperature tan δ peak became more distinct upon increasing the X_n of the PBA block, likely due to an increase in PBA domain width and domain spacing. In addition, the materials 3D printed using 28.2 wt% of PBA-CTA had a larger (higher integral) peak at 75 °C (related to *net*-P(AA-*stat*-PEGDA) phase) compared to the materials 3D printed at 43.9 wt% of PBA-CTA, while a larger (higher integral) peak at -35 °C (related to the PBA phase) was observed for the materials 3D printed at 43.9 wt% of PBA-CTA compared to 28.2 wt% of PBA-CTA. This aligns with the quantities of the reagents used in each system (Supplementary Table 2).

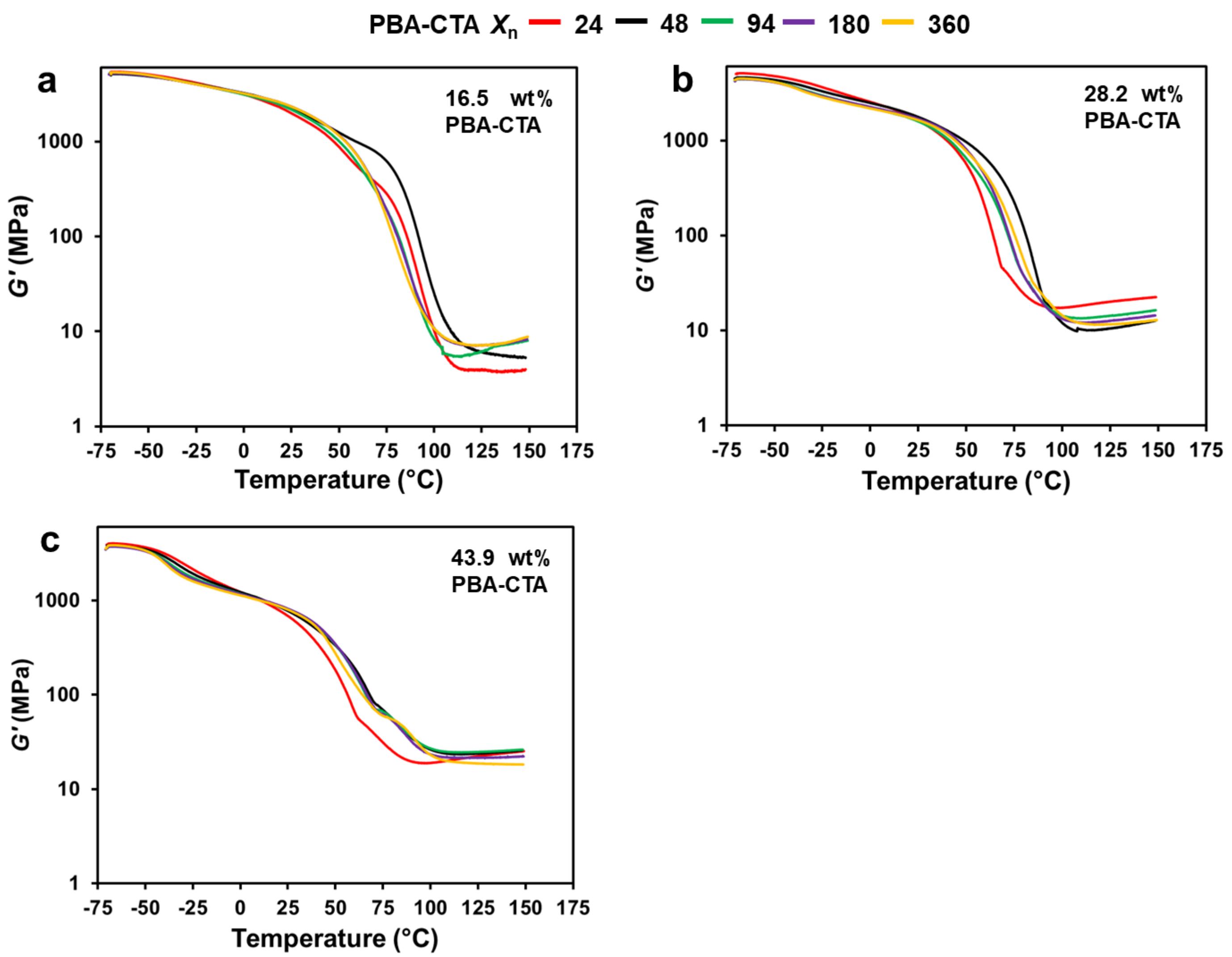

Supplementary Fig. 16. Storage modulus *G'* (MPa) in the temperature range of -70-150 °C for materials 3D printed using resins with various degree of polymerization (X_n) of PBA-CTA at three different weight percentage of PBA-CTA in a resin formulation: **a** 16.5 wt.%; **b** 28.2 wt%; **c** 43.9 wt%. Materials were 3D printed using a molar ratio of [AA]/[PEGDA] = 4/1. DMA was performed using a single cantilever bending test.

Supplementary Note 5: 3D printed materials displayed gradual softening with increasing temperature indicated by a drop in the storage modulus (*G'*). For materials 3D printed with 16.5 wt% of PBA-CTA, regardless of X_n of PBA-CTA *G'* gradually decreases from ~5200 MPa and materials soften around 100 °C (Supplementary Fig. 16a). Materials 3D printed with 28.2 and 43.9 wt% of PBA-CTA exhibited a stepwise drop in *G'* due to passing through the glass transitions of PBA and cross-linked PAA domains (Supplementary Fig. 16b-c).

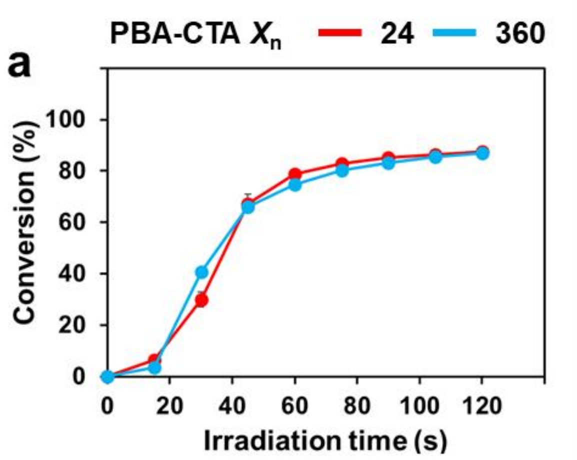

b

X_n of PBA-CTA	Tensile stress at break, σ_B (MPa)	Elongation at break, ε_B (%)	Toughness (MJ m^{-3})
24	38.7 ± 1.8	40.7 ± 4.9	13.3 ± 1.3
360	39.2 ± 1.3	63.0 ± 7.7	20.3 ± 3.5

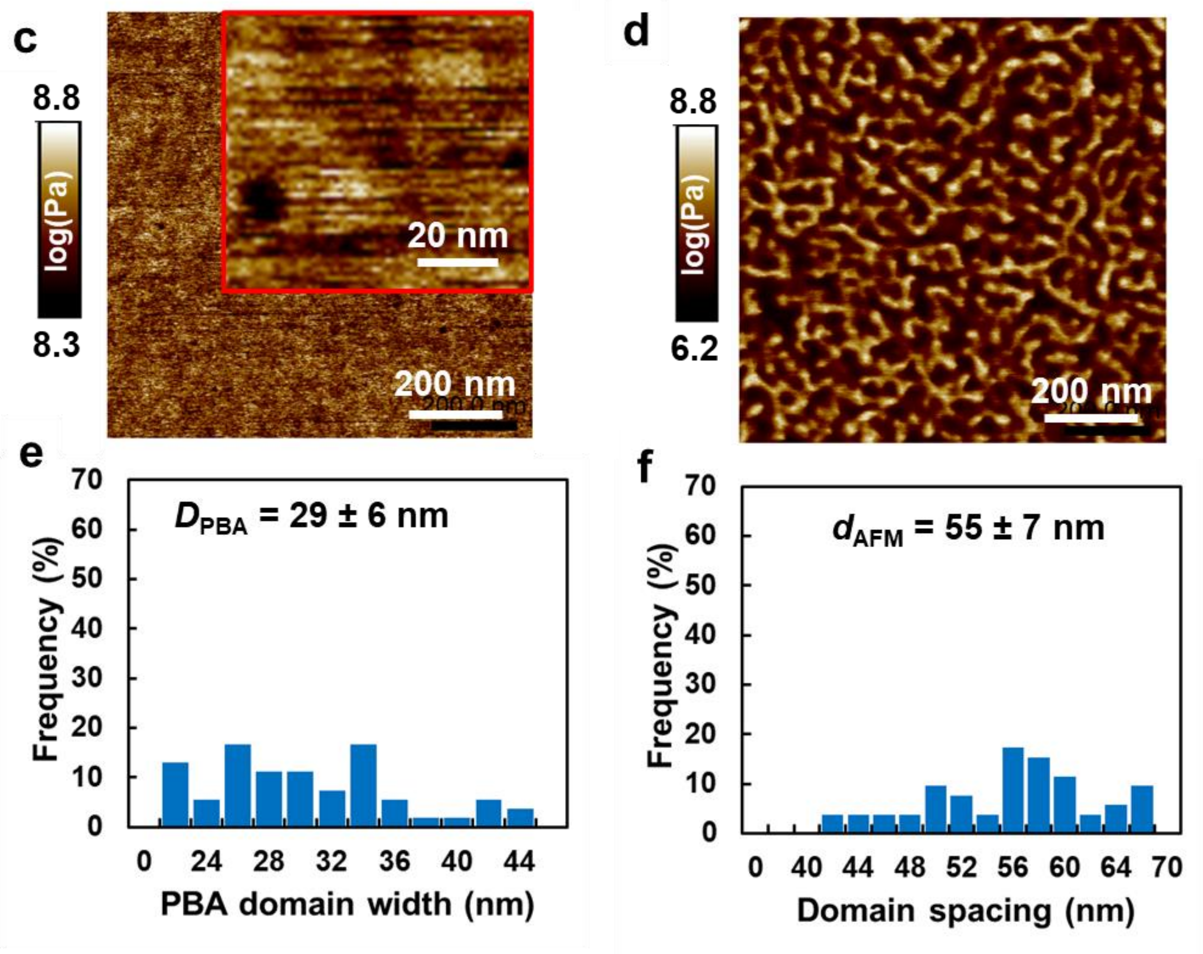

Supplementary Fig. 17. Comparison of 3D printed PIMS materials with different compositions but similar kinetic profiles. The resins with PBA-CTAs with X_n = 24 and 360 were formulated to produce **a** matching polymerization kinetics to study **b** mechanical properties and **c-d** surface morphologies of 3D printed samples, PBA-CTA X_n = (c) 24, (d) 360; **e** Average PBA domain width (D_{PBA}) and **f** domain spacing (d_{SAXS}) of material 3D printed with 16.5 wt% PBA_{360}-CTA. Materials were 3D printed using a molar ratio of [AA]/[PEGDA] = 4/1 at 16.5 wt% of PBA-CTA. Weight fractions of TPO were 0.3 and 0.133 wt% for PBA_{24}-CTA and PBA_{360}-CTA, respectively. Surface morphology was studied by AFM using PeakForce QNM. For **a** Error bars indicate standard deviation of at least duplicate measurements. Some error bars fall within the size of the markers. For **b** the data represents the mean ± s.d. of at least three independent experiments for each material.

Supplementary Table 5. Summary of morphology characterization and mechanical properties for 3D printed PIMS materials.

Resin #	PBA-CTA loading, wt%	X_n of PBA-CTA[a]	Morphology						Mechanical properties		
			Type[b]	D_{PBA}, (nm)[c]	d_{AFM}, (nm)[c]	L_{PBA}, (nm)[c]	d_{SAXS}, (nm)[d]		Tensile stress at break, σ_B (MPa)[e]	Elongation at break, ε_B (%)[f]	Toughness (MJ m^{-3})[g]
**1**	16.5	24	Globular domains	7 ± 2	-	-	11		38.7 ± 1.8	40.7 ± 4.9	13.3 ± 1.3
**2[h]**		48	Globular domains	9 ± 1	19 ± 3	-	16		40.7 ± 1.1	71.2 ± 4.6	24.9 ± 2.0
**3**		94	Elongated domains	13 ± 2	24 ± 2	92 ± 50	22		48.1 ± 2.1	91.4 ± 4.1	35.5 ± 2.6
**4**		180	Bicontinuous	17 ± 3	32 ± 3	-	33		49.2 ± 2.1	90.1 ± 3.7	35.7 ± 2.4
**5**		360	Bicontinuous	23 ± 2	55 ± 7	-	58		47.0 ± 1.6	82.2 ± 7.5	31.5 ± 2.9
**6**	28.2	24	Globular domains	7 ± 1	-	-	10		28.8 ± 2.0	60.2 ± 1.8	15.0 ± 1.7
**7[h]**		48	Globular domains	9 ± 1	17 ± 2	21 ± 11	14		31.2 ± 2.1	63.0 ± 4.2	16.7 ± 1.4
**8**		94	Bicontinuous	12 ± 2	23 ± 3	-	20		30.4 ± 0.6	77.0 ± 0.5	19.2 ± 0.5
**9**		180	Bicontinuous	15 ± 2	31 ± 4	-	29		27.9 ± 2.6	73.1 ± 3.3	16.5 ± 2.3
**10**		360	Bicontinuous	22 ± 4	45 ± 6	-	48		27.5 ± 0.6	58.5 ± 9.6	13.1 ± 2.3
**11**	43.9	24	Globular domains	7 ± 1	-	-	9		14.2 ± 0.6	82.2 ± 5.5	9.7 ± 1.4
**12[h]**		48	Globular domains	9 ± 2	15 ± 2	39 ± 20	13		15.2 ± 0.7	94.7 ± 0.9	11.7 ± 0.6
**13**		94	Bicontinuous	13 ± 2	21 ± 2	-	18		15.6 ± 1.2	99.5 ± 2.6	12.4 ± 1.3
**14**		180	Bicontinuous	15 ± 3	28 ± 3	-	26		14.0 ± 0.1	80.9 ± 1.1	9.1 ± 0.1
**15**		360	Bicontinuous	22 ± 4	43 ± 5	-	43		10.1 ± 0.9	55.0 ± 11.2	4.4 ± 1.2

[a] – Degree of polymerization (X_n) of PBA-CTA determined by ^{1}H NMR. [b] – Morphology of 3D printed materials determined by AFM. [c] – PBA domain width (D_{PBA}), PBA domain length (L_{PBA}) and domain spacing (d_{AFM}) determined from AFM. d_{AFM} values for X_n = 24 were not reported due to difficulty in precise measurement. [d] – Domain spacing (d_{SAXS}) determined from SAXS. [e] – Tensile stress at break was reported as the maximum tensile strength immediately before break. [f] – Elongation at break was reported as the maximum elongation of the sample immediately before break. [g] – Toughness was determined by calculating the area under a stress-strain curve using the trapezoidal rule. [h] – Please note that the mechanical properties of these 3D printed materials were reported in our previous work[7]. The data represents the mean ± s.d. of at least three independent experiments for each material.

Supplementary Table 6. Parameter values obtained from fitting of SAXS peaks using Teubner-Strey (T-S) model.

**wt% of PBA-CTA**	**X_n of PBA-CTA[a]**	**a_2[b]**	**c_1[b]**	**c_2[b]**	**d_{SAXS} (nm)[c]**	**d_{TS} (nm)[d]**	**ξ (nm)[e]**	**ξ/d_{TS}[f]**	**f_a[g]**
16.5	24	472.6	-2196.4	3095.4	11.1	10.3	7.5	0.73	-0.91
	48	530.2	-5050.7	14423.9	15.6	14.7	11.0	0.75	-0.91
	94	336.6	-6611.1	40888.2	22.2	21.4	14.2	0.66	-0.89
	180	275.9	-11673.7	159584.4	33.1	31.8	20.0	0.63	-0.88
	360	77.7	-7664.9	316508.6	58.0	53.3	23.7	0.45	-0.77
	360[h]	39.9	-4009.4	216070.3	66.0	58.7	21.6	0.37	-0.68
28.2	24	728.7	-3215.0	4136.1	10.1	9.9	8.0	0.81	-0.93
	48	626.4	-5288.6	13186.5	14.1	13.7	10.7	0.78	-0.92
	94	501.8	-8600.2	44425.0	19.6	19.7	14.5	0.74	-0.91
	180	288.8	-9896.1	108878.4	28.9	28.5	18.2	0.64	-0.88
	360	134.9	-10918.8	322366.8	48.3	45.9	23.8	0.52	-0.83
43.9	24	909.2	-3668.2	4244.4	9.5	9.4	8.1	0.86	-0.93
	48	873.3	-6784.4	15158.8	13.0	13.0	11.1	0.85	-0.93
	94	477.8	-7199.0	32852.4	18.4	18.5	13.5	0.73	-0.91
	180	421.9	-12461.9	112962.5	25.1	26.0	18.3	0.70	-0.90
	360	105.3	-6914.5	175098.7	44.5	42.2	20.5	0.48	-0.81

[a] – the degree of polymerization of PBA-CTA; [b] – parameters calculated from SAXS fitting using T-S model; [c] – domain spacing determined from SAXS; [d] – domain spacing determined from T-S fitting using Supplementary Equation (6); [e] – correlation length determined from T-S fitting using Supplementary Equation (7); [f] – The ratio of ξ/d_{TS} is a measure of the domain size polydispersity, the smaller the ratio, the larger the polydispersity[8]; [g] – amphiphilicity factor determined using Supplementary Equation (8). [h] – This PIMS material was 3D printed with lower amount of TPO (0.133 wt%).

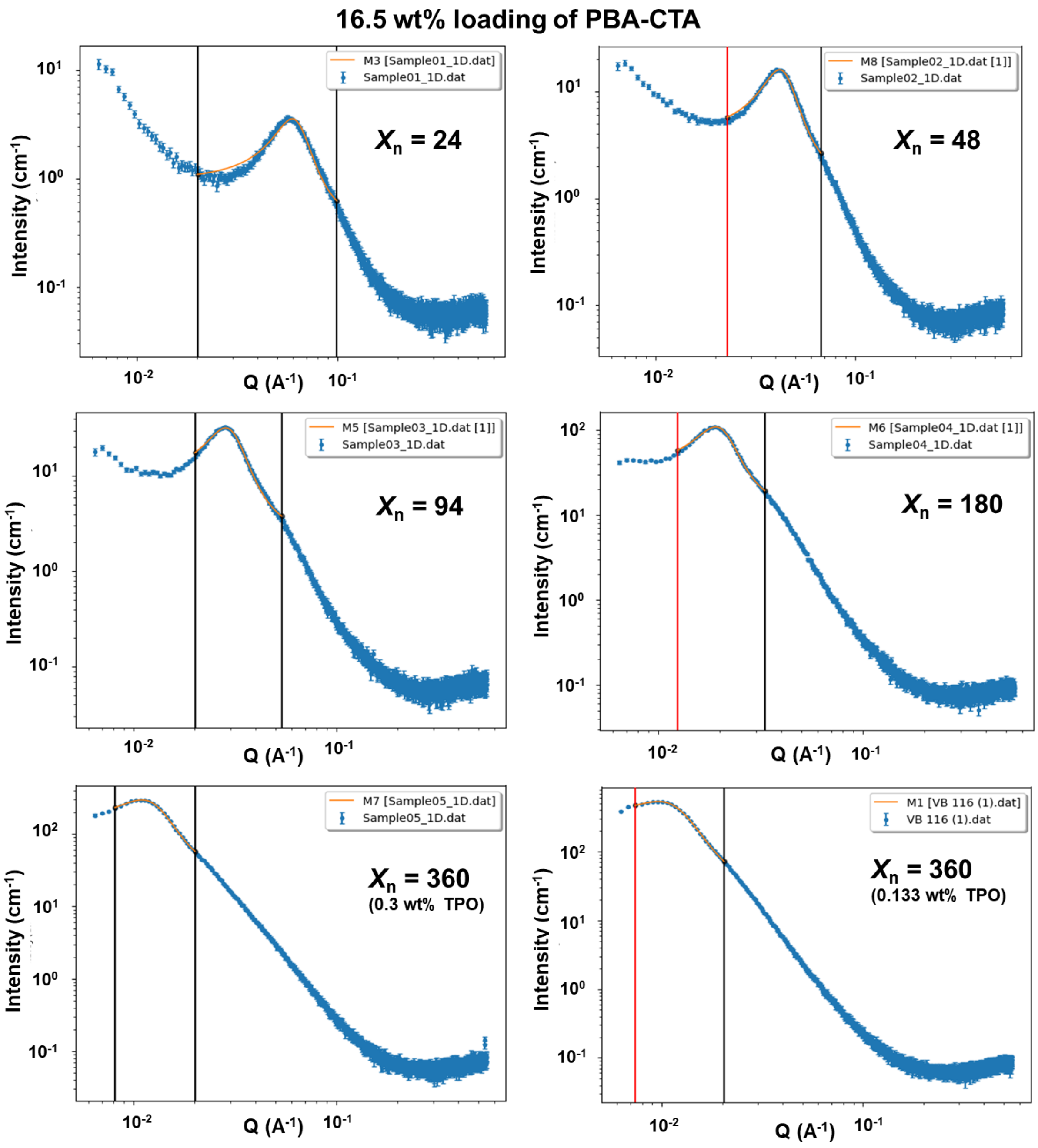

Supplementary Fig. 18. SAXS peaks fitted using Teubner-Strey (T-S) model for samples printed using 16.5 wt% PBA-CTA. The SAXS scattering data is presented as a blue curve and T-S fit is presented as an orange curve.

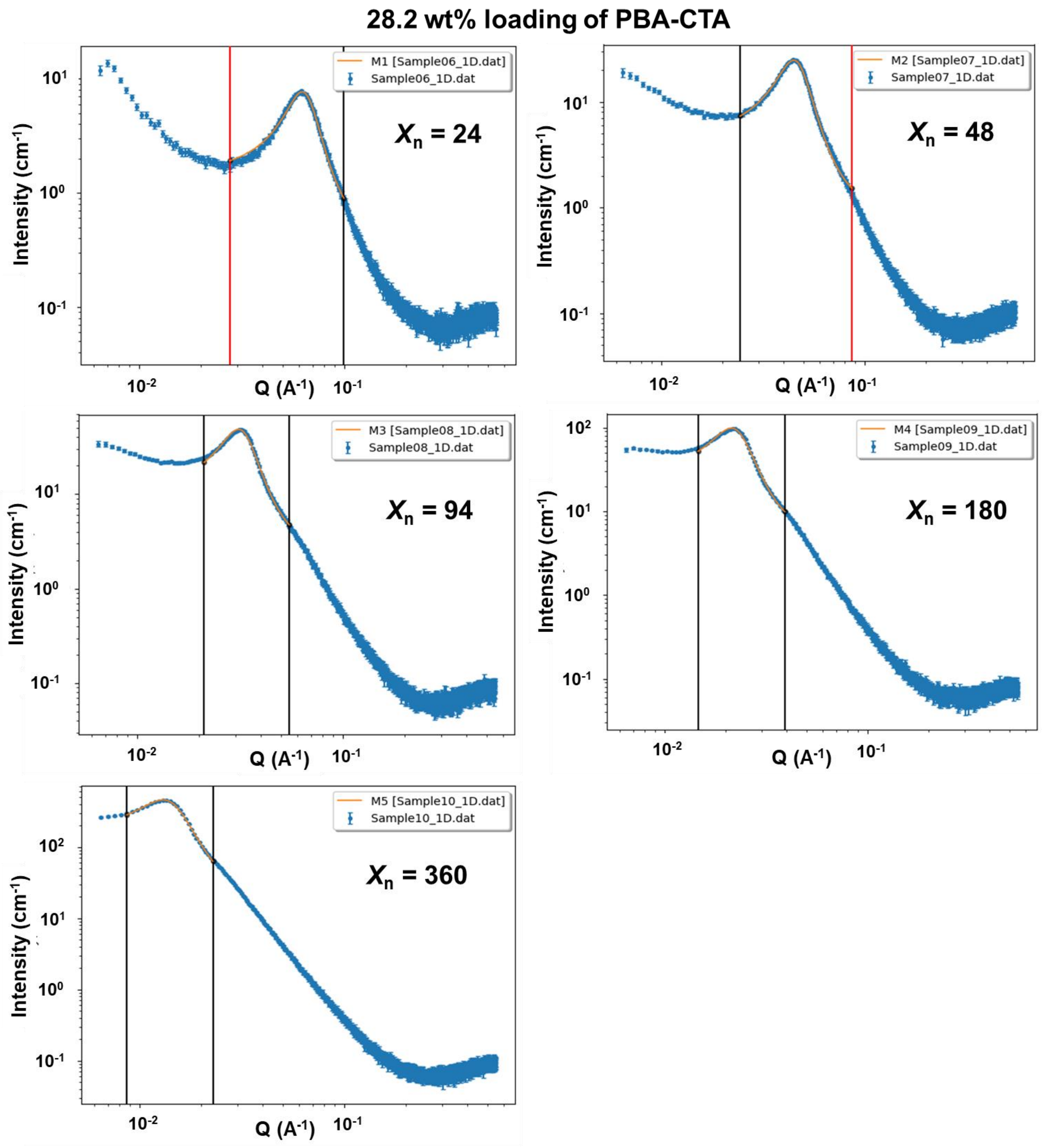

Supplementary Fig. 19. SAXS peaks fitted using Teubner-Strey (T-S) model for samples printed using 28.2 wt% PBA-CTA. The SAXS scattering data is presented as a blue curve and T-S fit is presented as an orange curve.

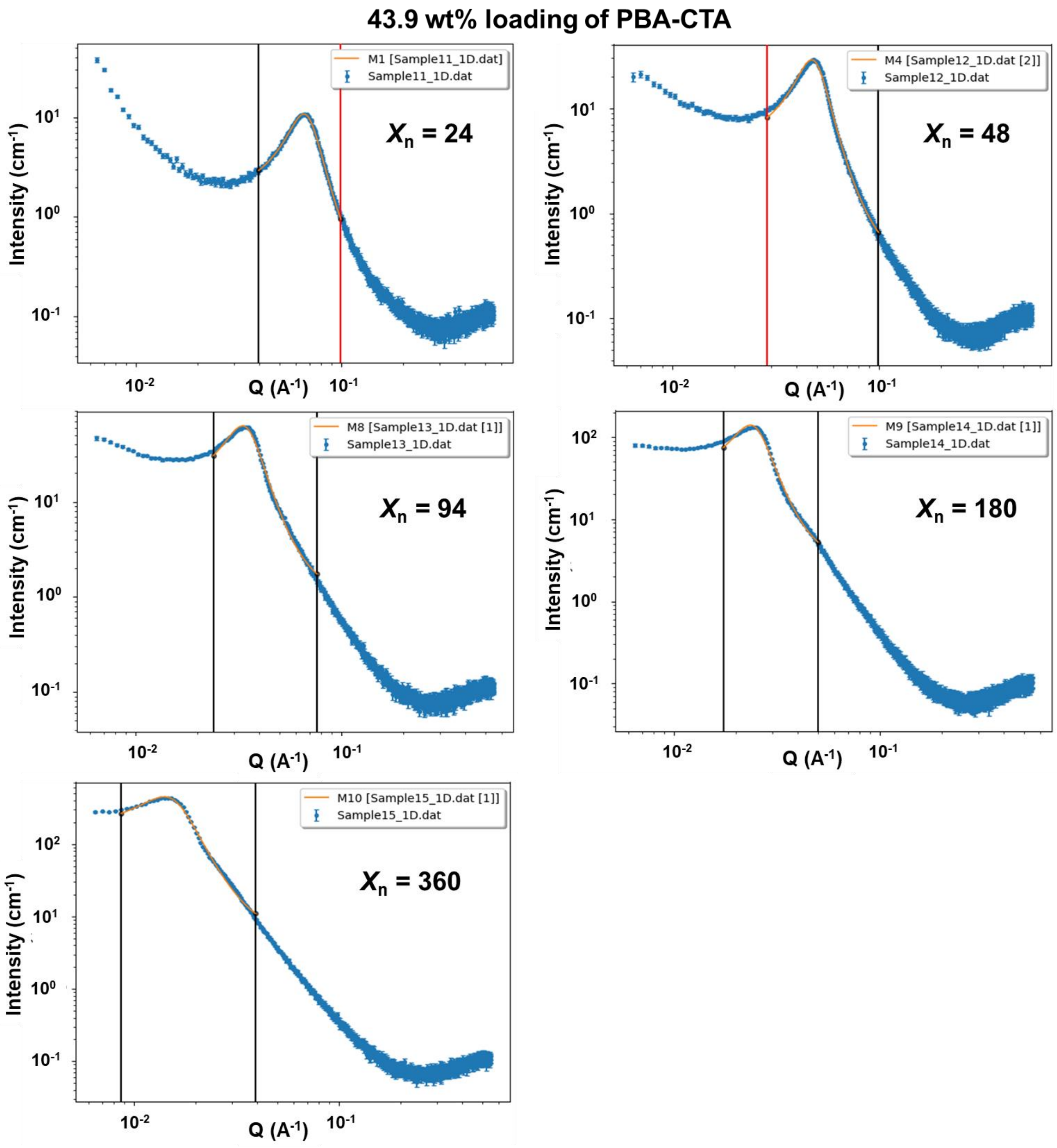

Supplementary Fig. 20. SAXS peaks fitted using Teubner-Strey (T-S) model for samples printed using 43.9 wt% PBA-CTA. The SAXS scattering data is presented as a blue curve and T-S fit is presented as an orange curve.

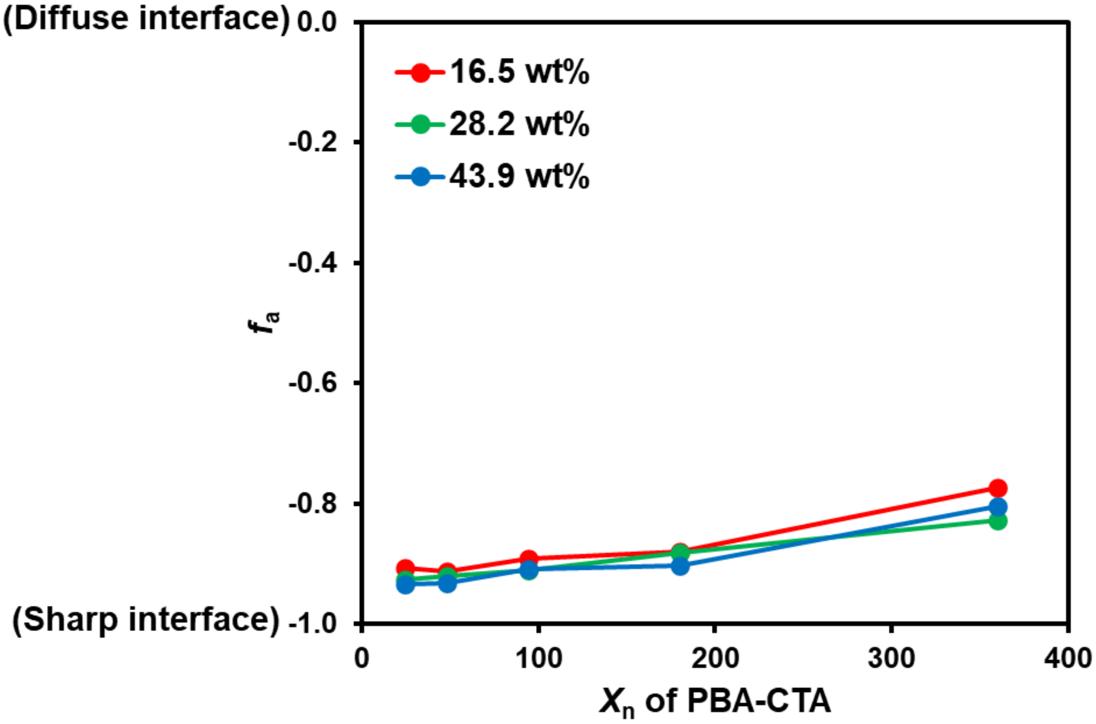

Supplementary Fig. 21. The amphiphilicity factor (f_a) as a function of the degree of polymerization (X_n) of PBA block.

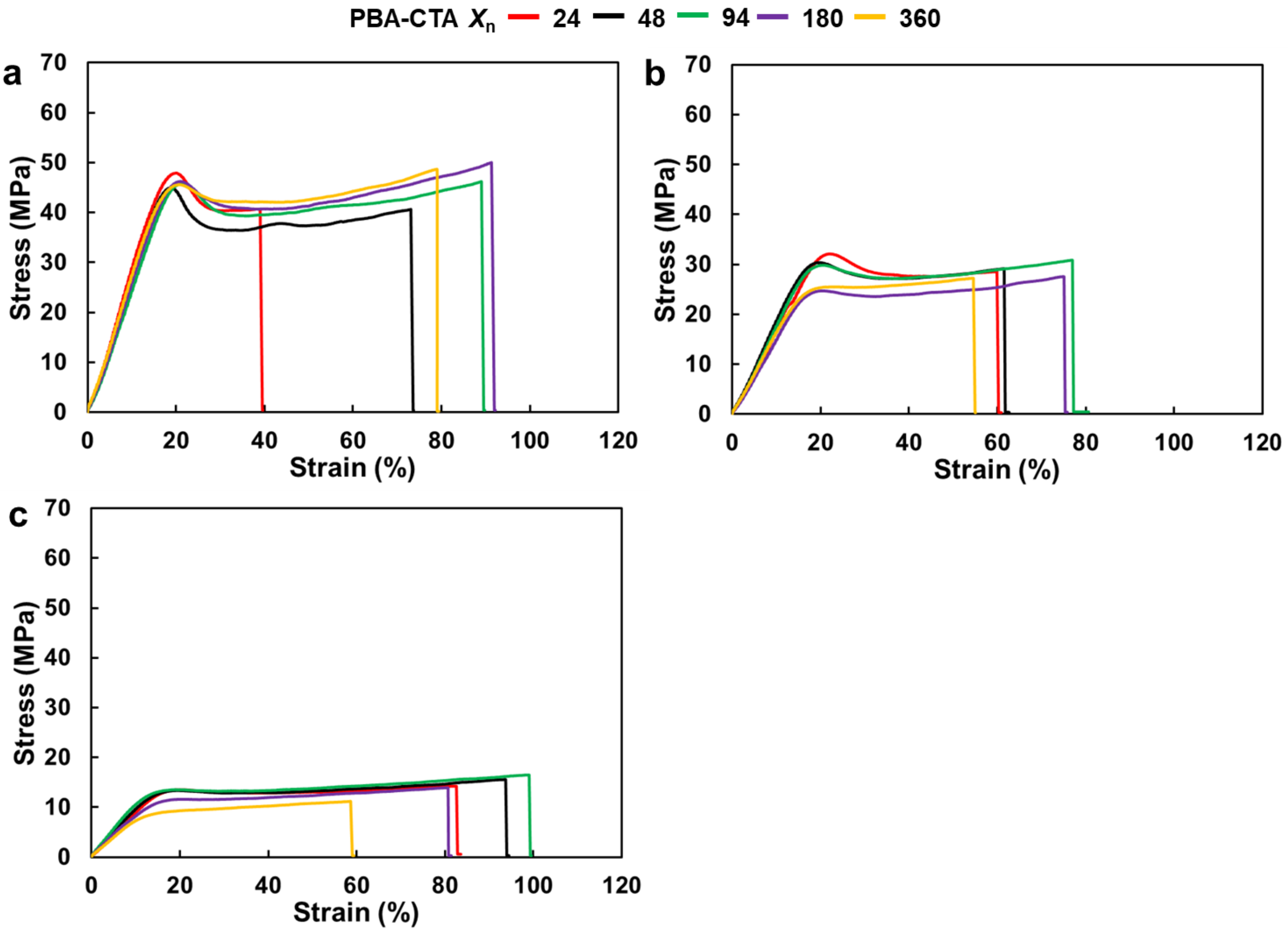

Supplementary Fig. 22. Representative stress–strain curves of samples 3D printed with various degree of polymerization (X_n) of PBA-CTA at three different weight percentage of PBA-CTA in a resin formulation: **a** 16.5 wt.%; **b** 28.2 wt%; **c** 43.9 wt%. Materials were 3D printed using a molar ratio of [AA]/[PEGDA] = 4/1.

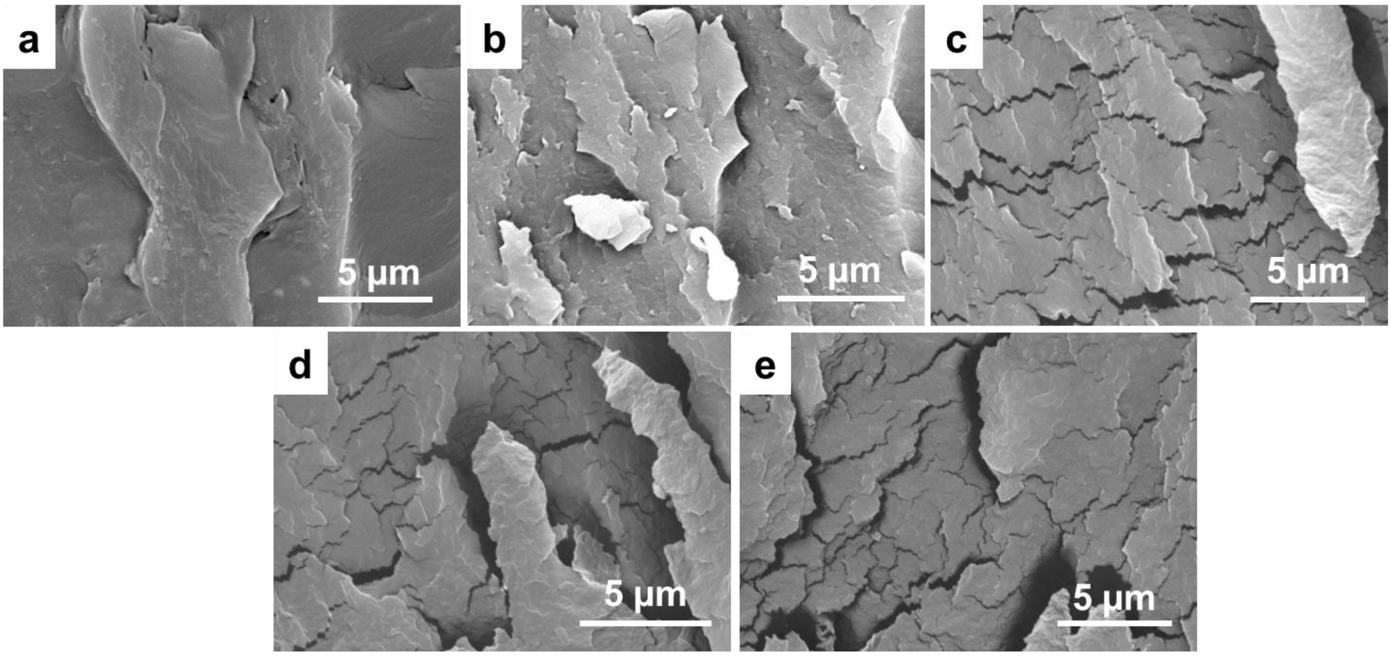

Supplementary Fig. 23. SEM fractography of fractured specimens from tensile testing. Materials 3D printed with various degree of polymerization (X_n) of PBA-CTA. **a** X_n = 24; **b** X_n = 48; **c** X_n = 94; **d** X_n = 180; **e** X_n = 360. Materials were 3D printed using a molar ratio of [AA]/[PEGDA] = 4/1 at 16.5 wt% of PBA-CTA.

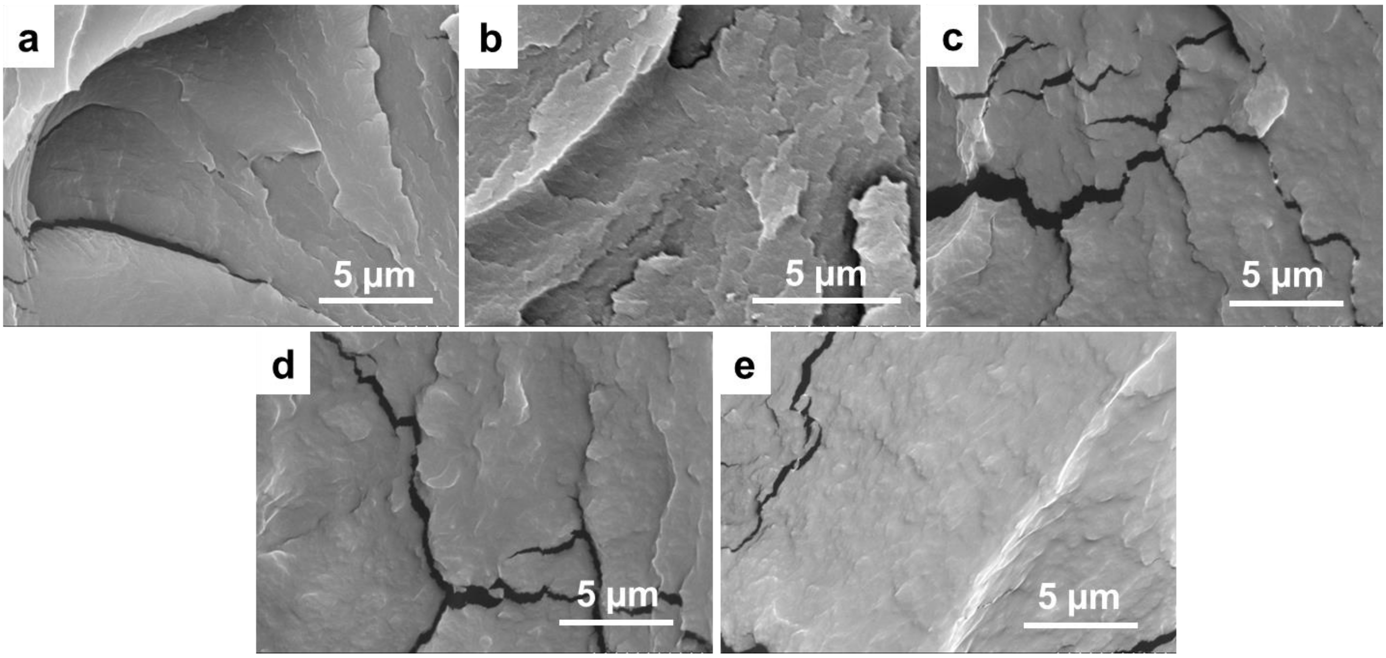

Supplementary Fig. 24. SEM fractography of fractured specimens from tensile testing. Materials 3D printed with various degree of polymerization (X_n) of PBA-CTA. **a** X_n = 24; **b** X_n = 48; **c** X_n = 94; **d** X_n = 180; **e** X_n = 360. Materials were 3D printed using a molar ratio of [AA]/[PEGDA] = 4/1 at 28.2 wt% of PBA-CTA.

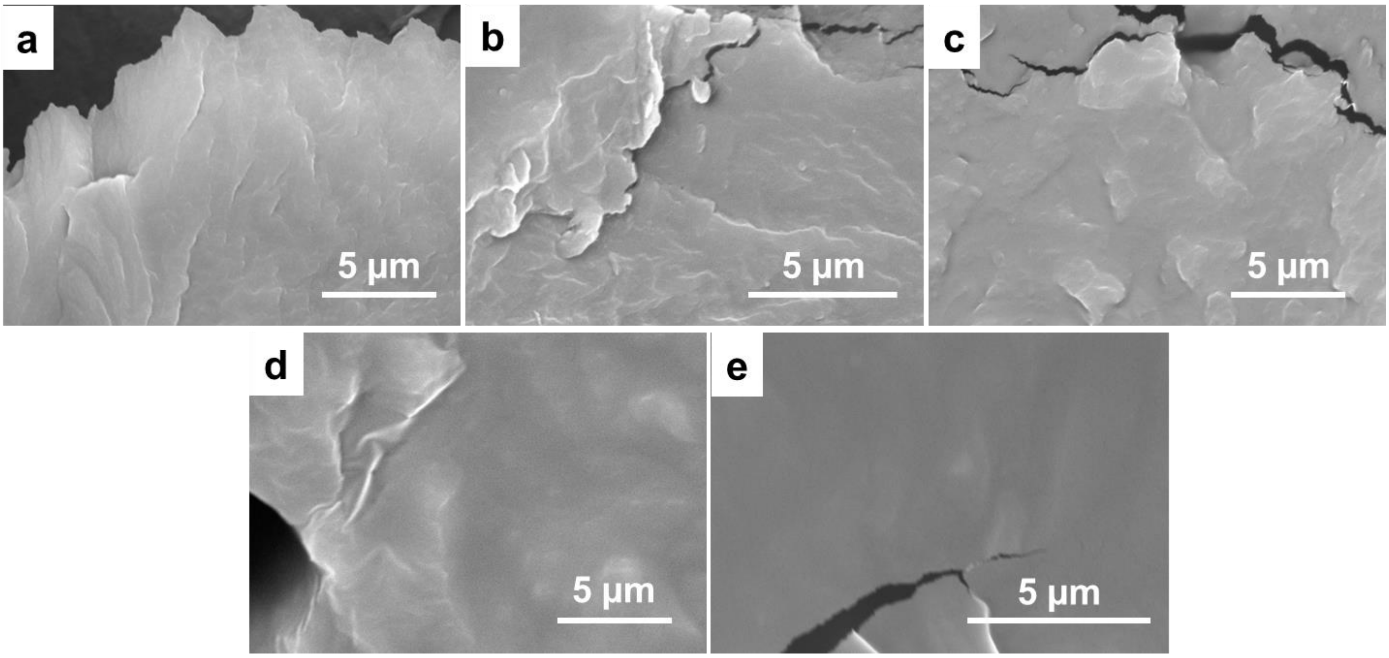

Supplementary Fig. 25. SEM fractography of fractured specimens from tensile testing. Materials 3D printed with various degree of polymerization (X_n) of PBA-CTA. **a** X_n = 24; **b** X_n = 48; **c** X_n = 94; **d** X_n = 180; **e** X_n = 360. Materials were 3D printed using a molar ratio of [AA]/[PEGDA] = 4/1 at 43.9 wt% of PBA-CTA.

Supplementary Note 6 (for Supplementary Figs. 23-25): For all three loadings of PBA-CTA, the fracture surface of 3D printed materials became rougher with the generation of microcracks upon increasing X_n of PBA block from 24 to 94 units (Supplementary Fig. 23a-c, Fig. 24a-c and Fig. 25a-c). For materials 3D printed with 16.5 wt% of PBA-CTA, further increase in X_n (180 and 360 units) resulted in no notable differences in fracture surface compared to X_n = 94, corresponding to no further enhancement in toughness (Supplementary Fig. 23d-e). For materials 3D printed with 28.2 and 43.9 wt% of PBA-CTA, the fracture surfaces with X_n = 180 and 360 were smoother with less microcracks compared to the fracture surface with X_n = 94 (Supplementary Fig. 24d-e and Fig. 25d-e). This corresponds to the lower toughness exhibited. Tougher materials absorb more fracture energy before cracking and have rougher fracture surface; crazing occurs in regions under high tension, or in regions of very localized yielding[9].

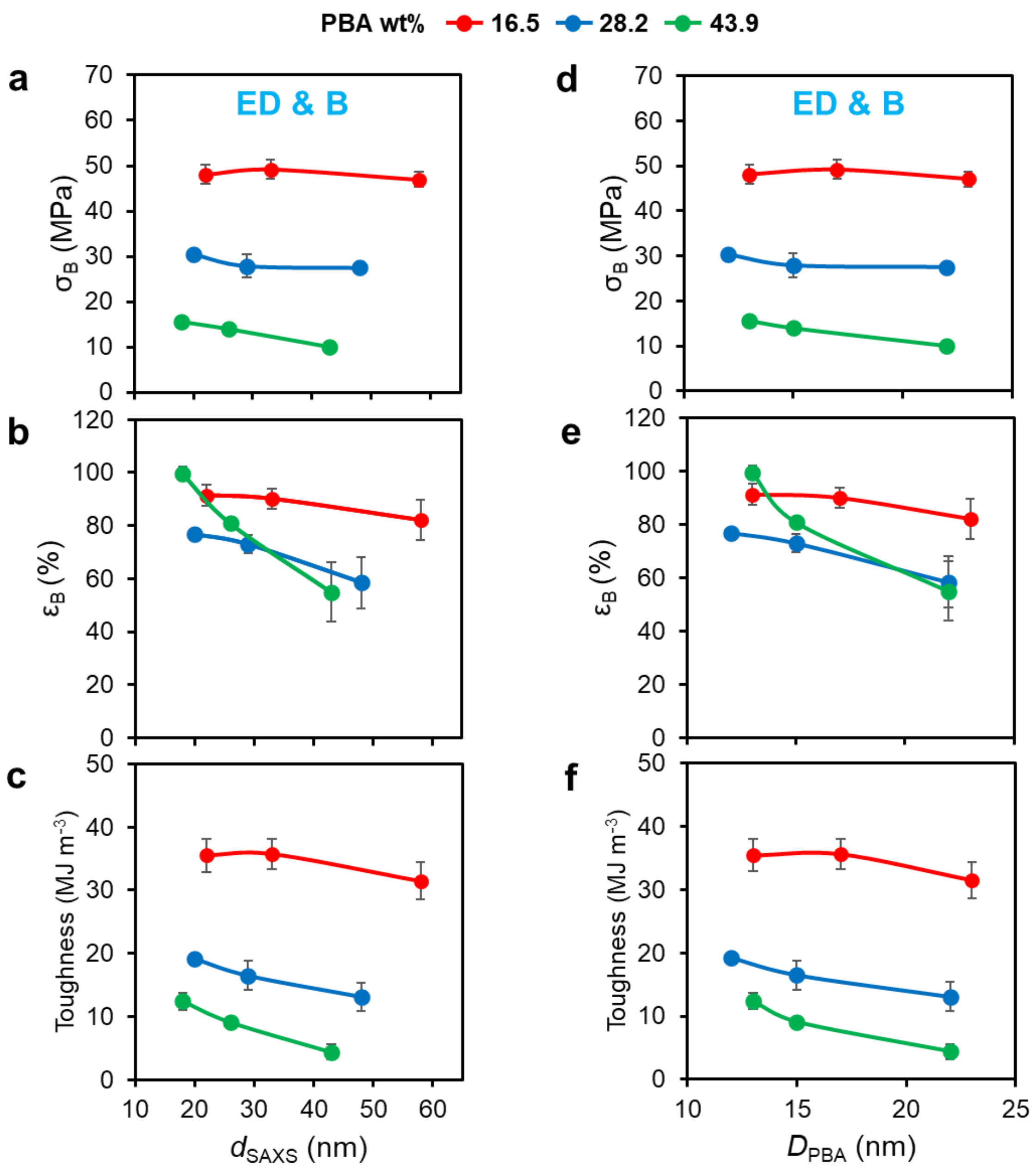

Supplementary Fig. 26. Mechanical property changes for materials with elongated domains and bicontinuous morphologies. Correlation of the tensile strength at break (σ_B), elongation at break (ε_B), and toughness of 3D printed samples with: **a-c** domain spacing (d_{SAXS}); **d-f** PBA domain width (D_{PBA}). Materials were 3D printed using a molar ratio of [AA]/[PEGDA] = 4/1 at a fixed PBA-CTA wt% of 16.5, 28.2 or 43.9 wt%. ED – elongated domains; B – bicontinuous morphology. Error bars indicate standard deviation in at least triplicate measurements. Some error bars fall within the size of the markers.

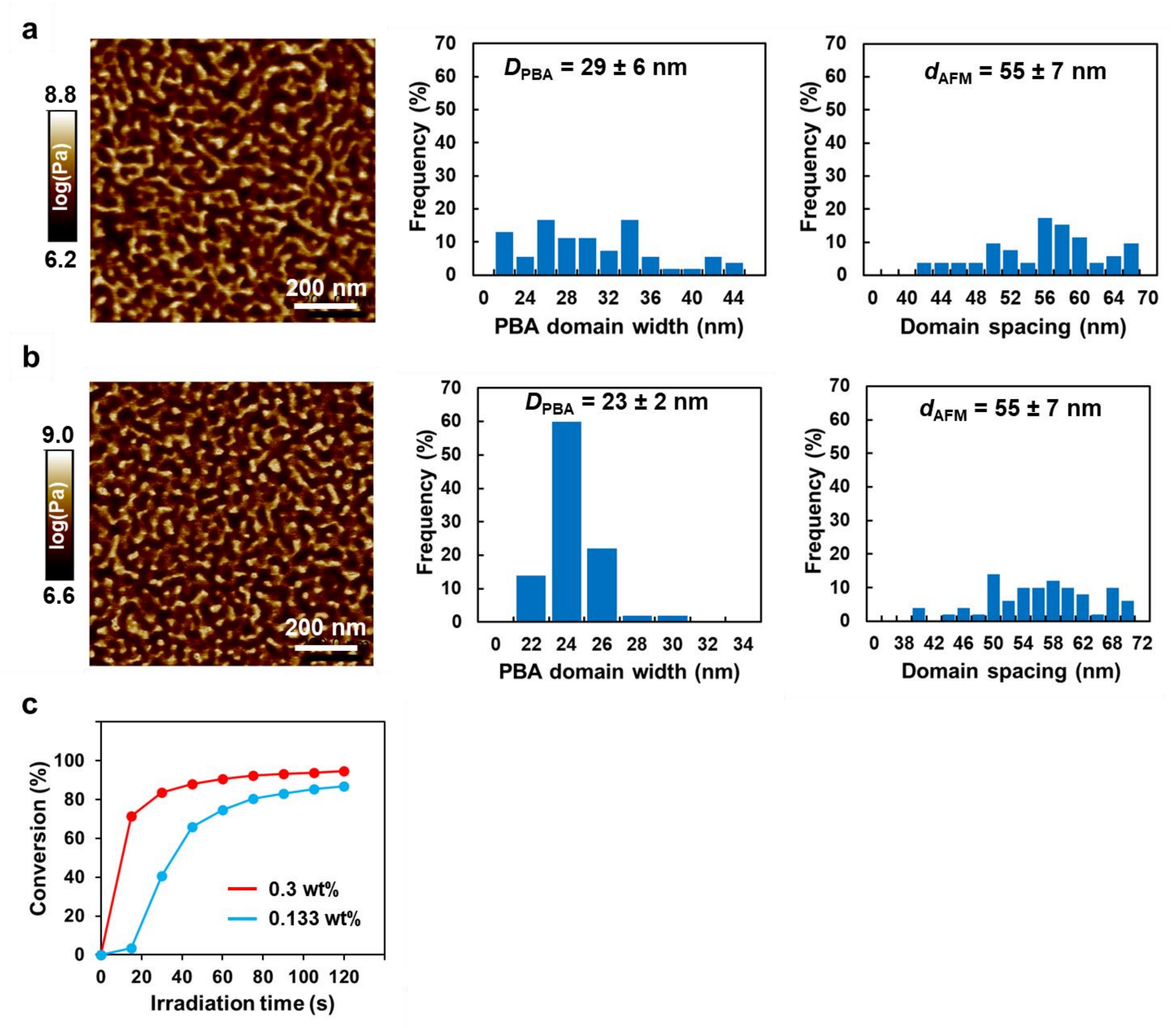

Supplementary Fig. 27. PeakForce QNM modulus map images and corresponding average PBA domain width (D_{PBA}) and domain spacing (d_{AFM}) of PIMS materials 3D printed using PBA_{360}-CTA at 16.5 wt% with **a** 0.133 wt% TPO, **b** 0.3 wt% TPO. **c** Polymerization kinetics of resins formulated with PBA_{360}-CTA (16.5 wt%) and 0.3 wt% TPO (red curve), 0.133 wt% TPO (blue curve). Error bars indicate standard deviation of at least duplicate measurements. Error bars fall within the size of the markers.

Supplementary Table 7. Summary of morphology characterization and mechanical properties for 3D printed PIMS materials with 16.5 wt% PBA_{360}-CTA and various wt% of TPO.

Resin #[a]	PBA-CTA loading, wt%	X_n of PBA-CTA[b]	TPO loading, wt%	Morphology					Mechanical properties		
				Type[c]	D_{PBA}, (nm)[d]	d_{AFM}, (nm)[d]	d_{SAXS}, (nm)[e]	f_a[f]	Tensile stress at break, σ_B (MPa)[g]	Elongation at break, ε_B (%)[h]	Toughness (MJ m^{-3})[i]
**5**	16.5	360	0.3	Bicontinuous	23 ± 2	55 ± 7	58	-0.77	47.0 ± 1.6	82.2 ± 7.5	31.5 ± 2.9
**16**			0.133		29 ± 6	55 ± 7	66	-0.68	39.2± 1.3	63.0 ± 7.7	20.3 ± 3.5

[a] – Please see Supplementary Table 2 for details of resin formulations. [b] – Degree of polymerization (X_n) of PBA-CTA determined by ^{1}H NMR. [c] – Morphology of 3D printed materials determined by AFM. [d] – PBA domain width (D_{PBA}) and domain spacing (d_{AFM}) determined from AFM. [e] – Domain spacing (d_{SAXS}) determined from SAXS. [f] – f_a values were determined from fitting of SAXS curves using Teubner-Strey (T-S) model. [g] – Tensile stress at break was reported as the maximum tensile strength immediately before break. [h] – Elongation at break was reported as the maximum elongation of the sample immediately before break. [i] – Toughness was determined by calculating the area under a stress-strain curve using the trapezoidal rule. The data represents the mean ± s.d. of at least three independent experiments for each material.

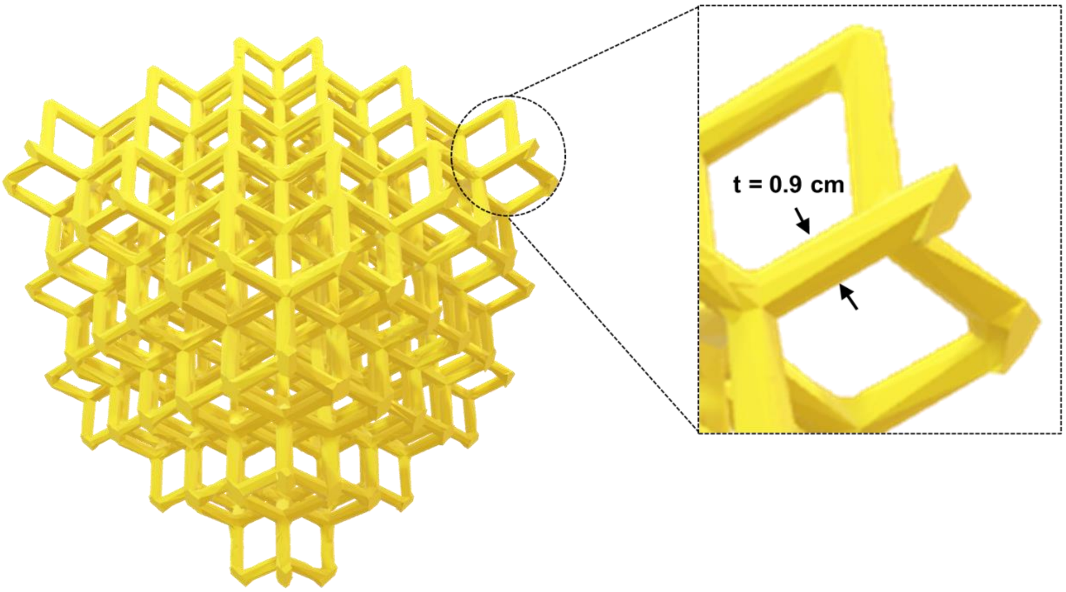

Supplementary Fig. 28. CAD model of 3D cubic lattice structure.

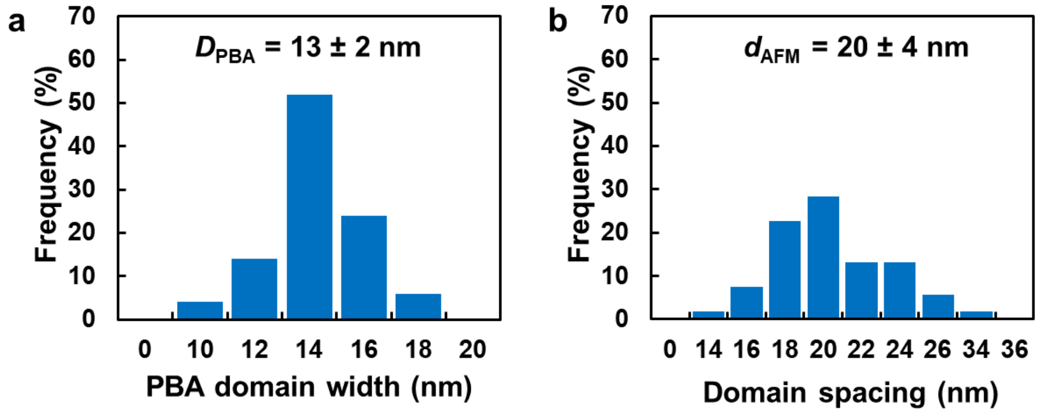

Supplementary Fig. 29. Average PBA domain width and domain spacing for the lattice structure 3D printed using PBA_{48}-CTA. **a** Domain width (D_{PBA}); **b** Domain spacing (d_{AFM}). The lattice structure was 3D printed using a molar ratio of [AA]/[PEGDA] = 4/1 at 28.2 wt% loading of PBA-CTA.

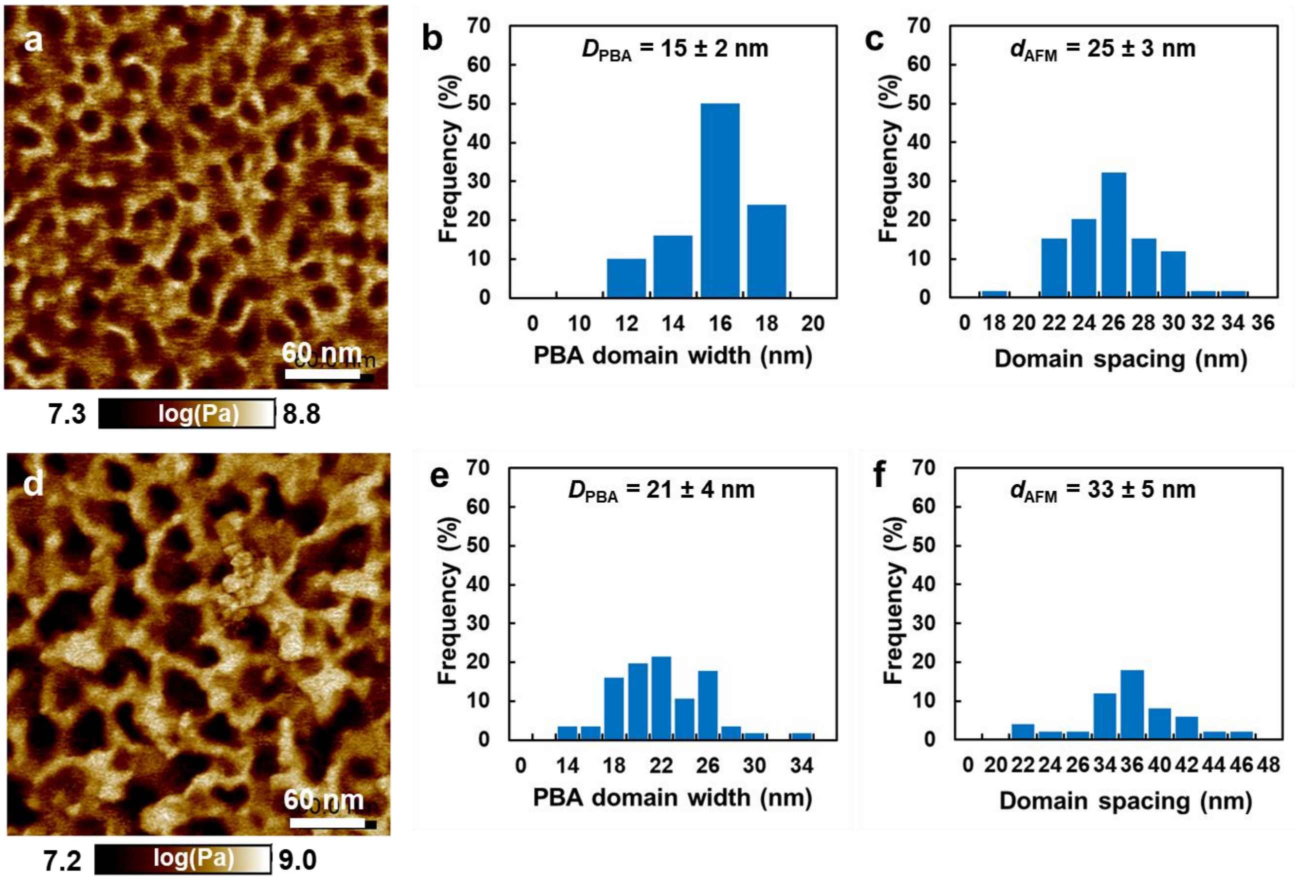

Supplementary Fig. 30. PeakForce QNM modulus map images and corresponding average PBA domain width (D_{PBA}) and domain spacing (d_{AFM}) of lattice structured 3D printed using PBA-CTA X_n = **a-c** 94, **d-f** 180 in Fig. 7 (main text).

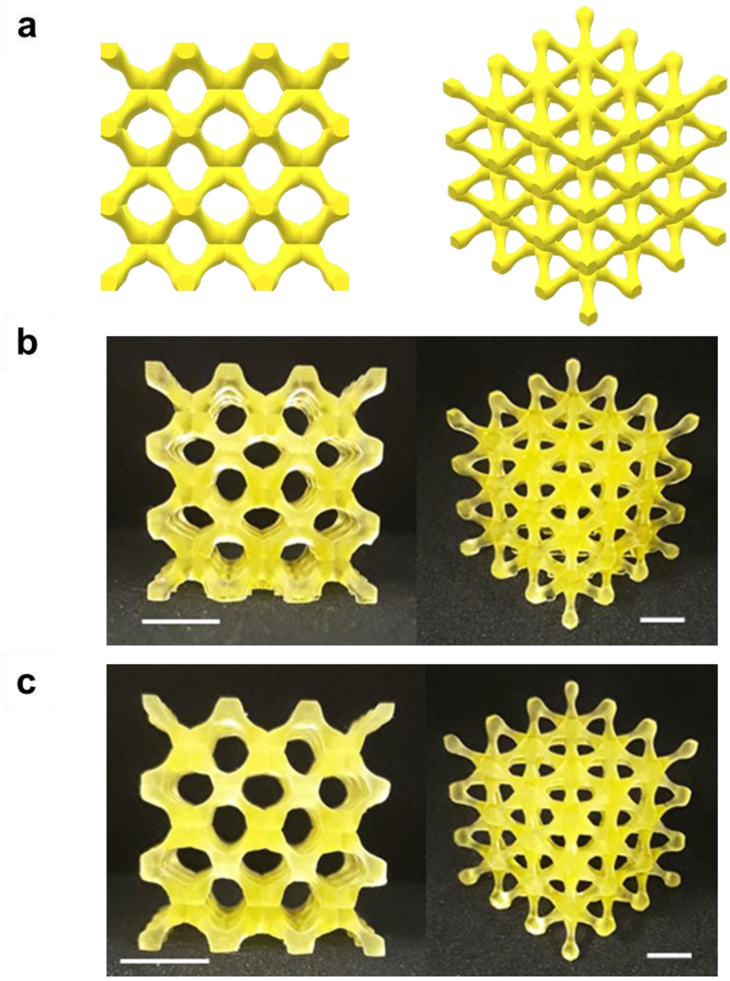

Supplementary Fig. 31. 3D printed lattice structures using PIMS and non-PIMS resins. **a** CAD model of body-centered lattice structure composed of the unit cells obtained using Bézier curve[10]; **b–c** BC lattice structures were 3D printed using **b** PIMS and **c** non-PIMS resins. The white line at the bottom represents a scale bar of 1 cm. PIMS resin formulation: 44.2 wt% AA, 38.4 wt% PEGDA, 16.4 wt% PBA_{48}-CTA, 1.0 wt% TPO. Non-PIMS resin formulation: 44.1 wt% AA, 38.3 wt% PEGDA, 16.0 wt% BA, 0.6 wt% BTPA, 1.0 wt% TPO.

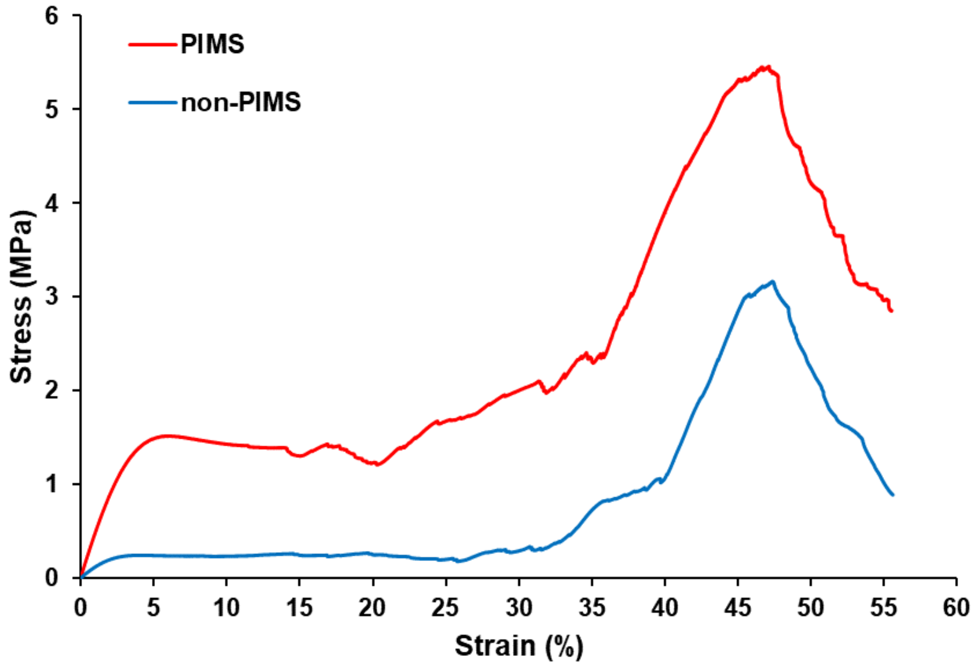

Supplementary Fig. 32. Representative stress-strain curves of the lattice structures 3D printed using PIMS and non-PIMS resins. PIMS resin formulation: 44.2 wt% AA, 38.4 wt% PEGDA, 16.4 wt% PBA_{48}-CTA, 1.0 wt% TPO. Non-PIMS resin formulation: 44.1 wt% AA, 38.3 wt% PEGDA, 16.0 wt% BA, 0.6 wt% BTPA, 1.0 wt% TPO.

Supplementary Table 8. Summary of mechanical properties obtained from compression testing of the lattice structures 3D printed using PIMS and non-PIMS resins.

Material[a]	Young's modulus (MPa)[b]	Strength (MPa)[c]	Modulus of resilience (kJ m^{-3})[d]	Toughness (MJ m^{-3})[e]
PIMS	0.4 ± 0.1	5.7 ± 0.5	12.2 ± 2.5	1.0 ± 0.2
Non-PIMS	0.1 ± 0.04	3.3 ± 0.3	1.2 ± 0.8	0.3 ± 0.05

[a] – PIMS material was 3D printed using the resin formulation: 44.2 wt% AA, 38.4 wt% PEGDA, 16.4 wt% PBA_{48}-CTA, 1.0 wt% TPO. Non-PIMS materials was 3D printed using the resin formulation: 44.1 wt% AA, 38.3 wt% PEGDA, 16.0 wt% BA, 0.6 wt% BTPA, 1.0 wt% TPO. [b] – Young's modulus was determined as a slope of the linear portion of the stress-strain curve within 0.1 – 0.2% of strain. [c] – Strength of the lattice structures was determined as a peak value of stress in the stress-strain curve. [d] – Modulus of resilience was determined as the total area under the stress-strain curve's linear elastic regime. It represents the material's maximum capacity to elastically absorb energy. [e] – Toughness was determined as the total area under the stress-strain curve and represents a material's maximum capacity to absorb energy. The total area under the stress-strain curve was calculated using the trapezoidal rule. The data represents the mean ± s.d. of three independent experiments for each material (PIMS and non-PIMS).

Supplementary References

1. https://www.atago.net/en/products-visco-top.php.

2. Teubner M, Strey R. Origin of the scattering peak in microemulsions. *The Journal of Chemical Physics* **87**, 3195-3200 (1987).

3. ASTM International, ASTM Standard D638-14: Standard Test Method for Tensile Properties of Plastics. (2014).

4. Small PA. Some factors affecting the solubility of polymers. *Journal of Applied Chemistry* **3**, 71-80 (1953).

5. Greenberg AR, Kusy RP. Influence of crosslinking on the glass transition of poly(acrylic acid). *Journal of Applied Polymer Science* **25**, 1785-1788 (1980).

6. Hasa E, Scholte JP, Jessop JLP, Stansbury JW, Guymon CA. Kinetically Controlled Photoinduced Phase Separation for Hybrid Radical/Cationic Systems. *Macromolecules* **52**, 2975-2986 (2019).

7. Bobrin VA, Lee K, Zhang J, Corrigan N, Boyer C. Nanostructure Control in 3D Printed Materials. *Advanced Materials* **34**, 2107643 (2022).

8. Chen SH, Chang, S. L., Strey, R. . On the Interpretation of scattering Peaks from Bicontinuous Microemulsions. *In Trends in Colloid and Interface Science IV; Zulauf, M, Lindner, P, Terech, P, Eds; Dr Dietrich Steinkopff Verlag* **81**, 30-35 (1990).

9. Zuppolini S, Zarrelli M, Zotti A. Chapter Fracture Toughening Mechanisms in Epoxy Adhesives in Adhesives: Applications and Properties (Ed.: A. Rudawska), InTech, Rijeka, Croatia.). InTechOpen (2016).

10. Lee S, Zhang Z, Gu GX. Generative machine learning algorithm for lattice structures with superior mechanical properties. *Materials Horizons* **9**, 952-960 (2022).
